# The role of Fe incorporation into Ni-MOF-74 derived oxygen evolution electrocatalysts for anion exchange membrane water electrolysis†

**DOI:** 10.1039/d4ey00250d

**Published:** 2025-02-04

**Authors:** Julia Linke, Thomas Rohrbach, Adam Hugh Clark, Camelia Borca, Thomas Huthwelker, Fabian Luca Buchauer, Mikkel Rykær Kraglund, Christodoulos Chatzichristodoulou, Eibhlin Meade, Julie Guehl, Mateusz Wojtas, Marco Ranocchiari, Thomas Justus Schmidt, Emiliana Fabbri

**Affiliations:** a PSI Center for Energy and Environmental Sciences 5232 Villigen PSI Switzerland emiliana.fabbri@psi.ch; b PSI Center for Photon Science 5232 Villigen PSI Switzerland; c Department of Energy Conversion and Storage, Technical University of Denmark Kongens Lyngby Denmark; d Institute of Molecular Physical Science, ETH Zürich 8093 Zürich Switzerland

## Abstract

The performance of Ni-based oxygen evolution reaction (OER) electrocatalysts is enhanced upon Fe incorporation into the structure during the synthesis process or electrochemical Fe uptake from the electrolyte. In light of the promising potential of metal–organic framework (MOF) electrocatalysts for water splitting, Ni-MOF-74 is used as a model catalyst to study the effect of Fe incorporation from KOH electrolyte on the electrocatalyst's OER activity and stability. The insights obtained from X-ray diffraction and operando X-ray absorption spectroscopy characterization of Ni-MOF-74 and an amorphous Ni metal organic compound (Ni-MOC*) reveal that Fe uptake enhances OER by two processes: higher Ni oxidation states and enhanced flexibility of both, the electronic state and the local structure, when cycling the potential below and above the OER onset. To demonstrate the impressive OER activity and stability in Fe containing KOH, an Ni-MOC* anode was implemented in an anion exchange membrane water electrolyzer (AEM-WE) with 3 ppm Fe containing 1 M KOH electrolyte resulting in an outstanding cell voltage of 1.7 V (at an anode potential of 1.51 V) at 60 °C and 0.5 A cm^−2^ exceeding 130 h of stable continuous operation.

## Abstract 2

The Fe uptake influence on Ni-MOF-74 derived oxygen evolution electrocatalysts is studied bridging operando XAS studies with implementation in AEM-WE.

## Graphical abstract

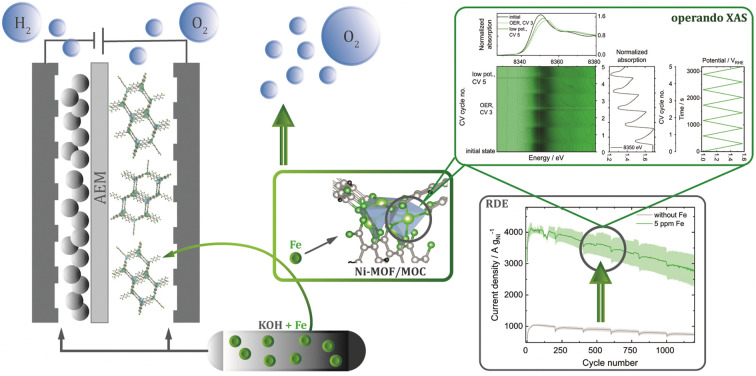

**Broader context**

Water electrolysis (WE) for hydrogen production can bridge energy demand and supply discrepancies or provide renewable hydrogen to decarbonize the chemical industry. A renewed focus on alkaline WE is driven by novel anion exchange membranes, which combine polymer-electrolyte cell setups with low-cost non-noble metal materials. Thereby, developing non-noble metal oxygen evolution reaction (OER) electrocatalysts is of great interest. Predominantly, Ni oxides doped with other transition metals are promising electrocatalysts for alkaline OER. Especially Fe incorporation – *via* Fe doping and Fe uptake from Fe-contaminated KOH electrolyte – results in impressive OER performances. Metal–organic framework (MOF) materials are alternative OER electrocatalysts, offering high porosity and tunable syntheses for tailored catalyst development. Herein, the Fe uptake from Fe-containing KOH electrolyte into Ni-MOF-74 and the resulting electronic and structural transformations are studied to simultaneously evaluate the MOF stability during OER and the advantageous Fe uptake into Ni-based MOF OER electrocatalysts. Additionally, a novel Ni-MOF derived catalyst with improved Fe incorporation and enhanced OER performance is tested in anion-exchange membrane WE. This comprehensive work provides key OER performance descriptors for the Ni-based OER electrocatalyst development and guidance for their application upon industrial conditions in Fe-contaminated KOH, resulting in a setup with outstanding performance and stability.

## Introduction

Among different electrolysis techniques, anion-exchange membrane water electrolysis combines various advantages^1–3^ and enables the use of non-noble metal electrocatalysts.^4–6^ The oxygen evolution reaction (OER) is one of the two reactions occurring during water electrolysis. As it suffers from slower kinetics than the hydrogen evolution reaction, research focuses on the development of improved non-noble metal OER electrocatalysts.^7,8^

The alkaline OER is traditionally catalyzed by Ni-based electrocatalysts.^9,10^ However, it is well recognized that the OER activity of NiO is outperformed by NiFe layered double hydroxide electrocatalysts,^7,11,12^ as Fe doping increases the OER performance.^13–15^ Additionally, the electron transfer is facilitated by Ni–O–Fe bonds creating spin channels.^16^ Apart from Fe-doping of Ni electrocatalysts, Fe incorporation into these electrocatalysts from the electrolyte has a similar performance-enhancing effect.^17–19^ Fe is a common impurity in commercial electrolyzers, originating from electrolyte impurities^20^ or steel tubing with possible contaminations of more than 2–3 ppm Fe.^21–23^ Hence, studying the concept of Fe incorporation from the electrolyte is of interest with regards to industrial application and the corresponding Fe effects in alkaline water electrolysis.^22,24^ The Fe uptake occurs as a dynamic equilibrium with reversible dissolution into the KOH electrolyte and re-incorporation of Fe into the Ni electrocatalyst.^25^ Hence, a well-defined Fe concentration within the electrolyte is important for studying the Fe incorporation mechanisms. Therefore, Fe-free KOH electrolyte is contaminated with a defined amount of Fe in the ppm–ppb range,^19,26,27^ whereby it is important to consider the Fe solubility limit at the distinctive temperature and KOH concentration.^28^ Chung *et al.*^29^ found that the increase in OER current of NiO_*x*_H_*y*_ is linearly correlated to the Fe surface coverage. However, once the maximum coverage is reached – which can already occur at low Fe concentrations of 0.1 ppm – a non-linear correlation of the OER activity enhancement with respect to the Fe atomic ratio in the electrolyte is observed, as the Fe adsorption saturates and an insulating FeOOH phase can be formed at high overpotentials.^29,30^ Even though there have been extensive studies of the Fe incorporation effect for several Ni oxide based catalysts, mechanistic studies of the Fe incorporation into novel Ni materials, as *e.g.*, metal organic frameworks (MOFs), highlighting the dynamic environment of the electrocatalytic interface, are needed. Additionally, the application of this activity enhancement in anion exchange membrane water electrolysis is crucial for a successful transition into industrial application.

Recently, MOFs have been studied for alternative clean energy carrier production to replace fossil fuels, particularly advancing sustainable hydrogen production as electrocatalysts in water electrolysis.^6^ Metal organic frameworks (MOFs) consist of organic linkers that connect metal nodes to form a porous structure.^5^ This building-block concept offers a great variability to tailor the syntheses in terms of the material's porosity and conductivity. Due to this structural tunability, the interest in the application of MOFs has increased significantly in recent years,^4,5,31,32^ including the use for hydrogen storage and electrocatalysis.

Herein, the transformations of a Ni-based MOF catalyst (Ni-MOF-74) upon Fe-incorporation during OER are analyzed, as Fe uptake into Ni OER catalysts enhances their performance. The monitored catalyst's restructuring was used as a model for the novel synthesis development of an amorphous Ni metal organic compound (Ni-MOC*) catalyst with unprecedented OER activity in Fe containing electrolyte. Application of this novel electrocatalyst as the anode during anion-exchange membrane water electrolysis with 3 ppm Fe contaminated KOH electrolyte shows a remarkable performance during 130 h of operation at 500 mA cm^−2^.

## Results and discussion

### Structural and electrocatalytic evaluation of Ni-MOF-74 upon Fe uptake

**Fig. 1:**
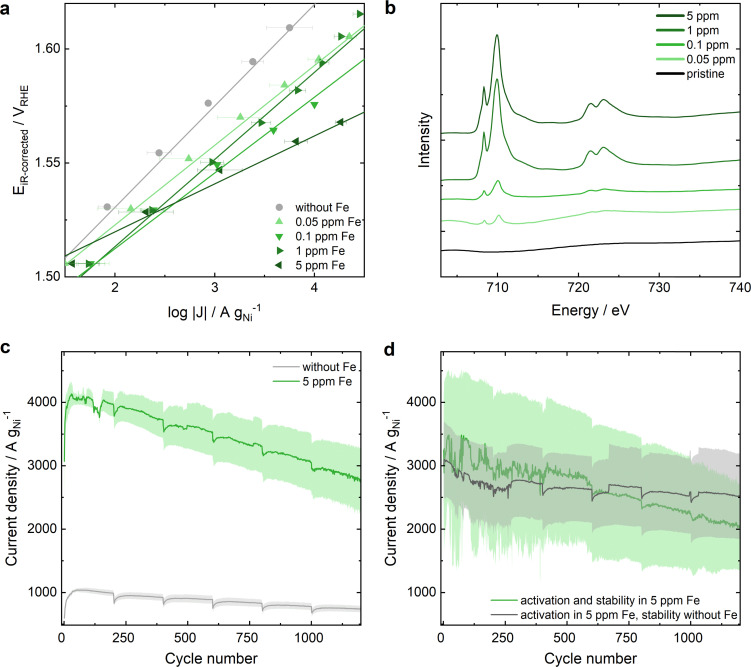
OER performance of Ni-MOF-74 and its Fe uptake from Fe-containing 0.1 M KOH. (a) Tafel plots of Ni-MOF-74 extracted from chronoamperometric step measurements from 1 to 1.7 *V*_RHE_ in KOH with varying Fe concentrations of 0 to 5 ppm. The error bars present the standard error after averaging 3 measurements. (b) sXAS of Fe L edge of pristine Ni-MOF-74 and Ni-MOF* samples after OER in 0.1 M KOH containing different Fe ratios. (c) Comparison of the electrochemical stability of Ni-MOF-74 in Fe-free and 5 ppm Fe contaminated KOH (detailed measurement protocol in ESI†). (d) Electrochemical stability of Ni-MOF-74 after the activation in 5 ppm Fe containing KOH: Stability in 5 ppm Fe KOH (green) compared to the stability in Fe-free KOH (grey).

In Ni-MOF-74, the 2,5-dioxidotherephthalate linker connects the Ni metal nodes that form hexagonal 1D channels (structure shown in Fig. S1, ESI†). To confirm a successful synthesis, Ni-MOF-74 was characterized by powder X-ray diffraction (PXRD), attenuated total reflection infrared spectroscopy (ATR-IR), Brunauer–Emmett–Teller (BET) surface area analysis and transmission electron microscopy (TEM) (Fig. S2 and S3, ESI†). A three-electrode rotating disc electrode (RDE) cell was adopted to study the OER performance of the electrocatalyst in 0.1 M KOH (protocol details in ESI†). Chronoamperometric measurements were carried out from 1.2 *V*_RHE_ to 1.7 *V*_RHE_ – after 25 cyclic voltammograms (CVs) for equilibration (Fig. S4, ESI†) – to extract the Tafel plots shown in Fig. 1a. In Fe-free KOH (grey dots, see ESI† for electrolyte specifications) a remarkable activity of approximately 273 A g_Ni_^−1^ is achieved at 1.55 *V*_RHE_ with a Tafel slope of 42 mV dec^−1^. Increasing Fe contamination results in an enhanced OER activity of up to approximately 1350 A g_Ni_^−1^ at 1.55 *V*_RHE_ in 0.1 ppm Fe containing 0.1 M KOH with a decrease in the Tafel slope down to 33 mV dec^−1^. Increasing the Fe contamination further to 1 ppm leads to a decrease in OER activity. A maximum activity at an Fe concentration of 0.1 ppm Fe in KOH electrolyte is in agreement with the study of Chung *et al.*^29^ that reports a maximum Fe surface coverage for NiO_*x*_H_*y*_ at 0.1 ppm Fe in 0.1 M KOH. However, for Ni-MOF-74 a further increase to 5 ppm Fe results in a further change of the Tafel slope down to 21 mV dec^−1^ and in the highest OER activity recorded for Ni-MOF-74 with 2700 A g_Ni_^−1^ at 1.55 *V*_RHE_. The different Tafel slope indicates a change in the rate determining step of the reaction mechanism^33–35^ dominating over the Fe surface coverage influence. For comparison, the OER performance of commercial NiO in Fe-free and 5 ppm Fe containing KOH are depicted in Fig. S5 (ESI†) (20.2 A g_Ni_^−1^ at 1.55 *V*_RHE_ in Fe-free KOH and 330 A g_Ni_^−1^ at 1.55 *V*_RHE_ in 5 ppm Fe containing KOH).

Soft X-ray absorption (sXAS) measurements at the Fe L edges were conducted to qualitatively determine the Fe content at the near-surface region of the Ni-MOF-74 catalyst after electrochemical reaction in Fe-containing KOH, referred to as Ni-MOF*. The results in Fig. 1b show a larger Fe content at the surface-near region of the catalyst (5–10 nm depth) after electrochemical testing in KOH electrolyte with increasing Fe concentration. The depicted Fe L edges show similar peak ratios to the reference material Fe_2_O_3_,^36,37^ indicating the presence of Fe^3+^ on the surface of Ni-MOF*. As both the 1 ppm and 5 ppm samples have higher Fe surface concentrations compared to 0.1 ppm, while one of them performs worse than 0.1 ppm and the other outperforms it, it can be concluded that the Fe content at the surface-near region of the catalyst is not the only significant factor determining the OER activity increase.

The OER stability is studied by reversible stepwise cycling of the applied potential between 1 *V*_RHE_ and 1.6 *V*_RHE_. Thereby, the current density monitored at 1.6 *V*_RHE_ is 4 times higher in 5 ppm Fe containing KOH compared to Fe-free KOH indicating a higher OER activity, but a larger decrease in current is noted expressing an inferior stability (Fig. 1c). However, at the end of the stability test after 7 h of operation the activity is still 2.5 times higher in Fe-containing KOH. The inferior stability in 5 ppm Fe electrolyte is in line with the above-described phenomenon of an unfavorably high Fe uptake resulting in a lower activity. Hence, better stability was reached when doing an initial activation in 5 ppm Fe containing KOH, followed by the stability measurement protocol (specified in ESI†) in Fe-free KOH (grey curve in Fig. 1d), compared to activation and stability in 5 ppm Fe KOH (green curve, Fig. 1d). Different variations of Fe activation and stability testing are further elaborated in Fig. S6 (ESI†), emphasizing the necessity of a balanced amount of Fe incorporation.

### Transformations of Ni-MOF-74 during CV cycling in Fe-containing electrolyte

**Fig. 2:**
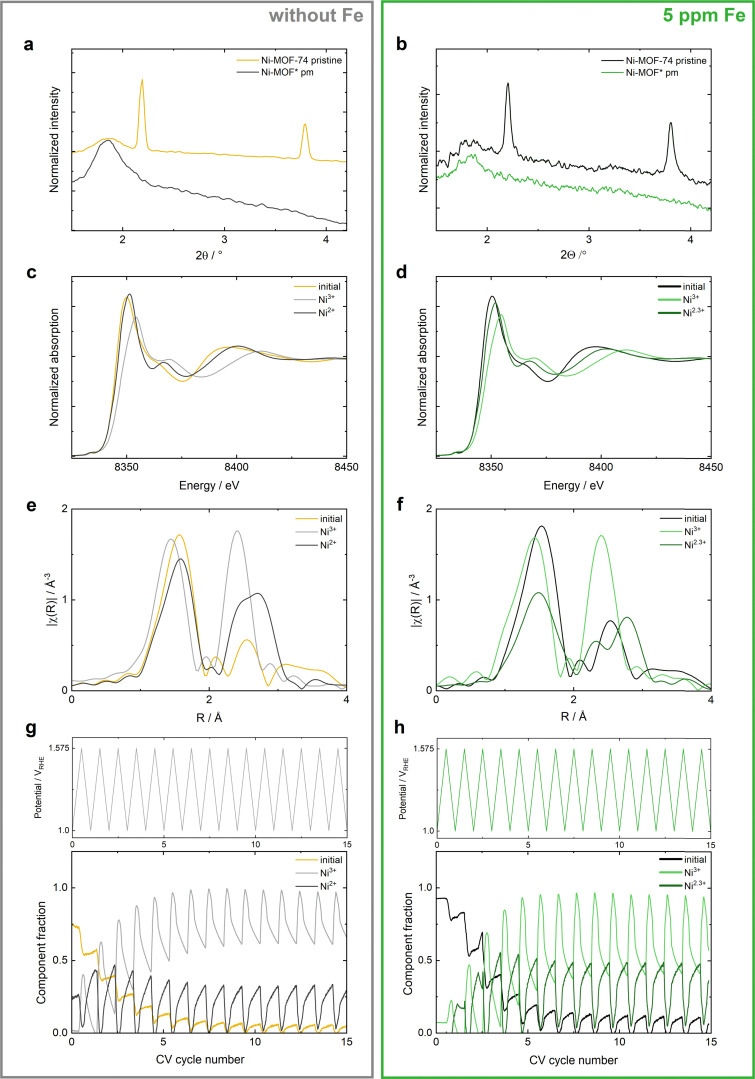
*Ex situ* XRD and operando hXAS measurements of Ni-MOF-74 in Fe-free (left side) and 5 ppm Fe contaminated (right side) 0.1 M KOH electrolyte. (a) and (b) *Ex situ* XRD of pristine Ni-MOF-74 before the electrochemical reaction and postmortem (pm) Ni-MOF* after OER. (c) and (d) XANES of the 3 components obtained by MCR analysis of the results obtained over the whole measurement duration of 15 CV cycles. (e) and (f) Fourier-transformed *k*^2^-weighted EXAFS of the 3 MCR components extracted from 15 CV cycles. (g) and (h) Component fractions of the three MCR components recorded over the measurement duration of 15 CV cycles. The oxidation state determination and the LOF of the MCR fitting are specified by Linke *et al.*^38^ for Fe-free KOH and in Fig. S10 and S11 (ESI†) for Fe-KOH.

To study the structural transformations induced by the Fe incorporation of Ni-MOF-74, *ex situ* XRD was conducted after electrochemical testing in KOH with and without Fe. For comparison, the XRD characterizations of commercial NiO are listed in Fig. S7 (ESI†). The XRD measurements of Ni-MOF-74 in Fig. 2a and b confirm the prior discussed destruction of the MOF-74 framework during OER^38^ in both Fe-free and 5 ppm Fe containing KOH. After OER, an amorphous highly OER active species (Ni-MOF*) derived from Ni-MOF-74 is obtained.

This restructuring of the MOF-74 framework into a highly OER active amorphous Ni electrocatalyst is analyzed in detail with time-resolved operando hard-XAS (hXAS) at the Ni K edge (Fig. 2c–h). It is very important to differentiate between the effect of Ni restructuring and Fe incorporation on the OER activity by performing electrochemical characterizations and operando XAS measurements in both, Fe-free and Fe-containing KOH electrolyte. Operando hXAS measurements were therefore conducted both in Fe-free and 5 ppm Fe containing KOH and can be compared to the operando hXAS results obtained for commercial NiO in Fig. S8 (ESI†). A resolution of 10 mV per XAS spectrum is achieved in continuous hXAS measurements during consecutive CV cycles from 1 *V*_RHE_ to 1.575 *V*_RHE_ at a scan rate of 2 mV s^−1^. The detailed protocol is explained in the ESI† and exemplified in prior publications.^38^ The material's stability under the beam and in the KOH electrolyte without performing OER is stated in Fig. S9 (ESI†).

Overall, multivariate curve resolution (MCR) revealed 3 main components for both conditions (Fig. 2c–h, oxidation state determination and lack of fit are specified in Fig. S8 and S9, ESI†): The *initial* component for the pristine Ni-MOF-74, a Ni^3+^ component at high potentials and a reduced Ni^2+/2.3+^ component appearing after the first 1–2 CV cycles. Both, the *initial* and the Ni^3+^ state are very similar in Fe-free and 5 ppm Fe contaminated KOH. However, a more pronounced splitting of the first shell (Ni–O) of the Ni^3+^ component is detected in Fe-KOH (Fig. 2f). In both electrolytes, the initial state vanishes within the first approximately 6 CV cycles during OER (Fig. 2g and h). In contrast, the Ni^3+^ component is formed at applied potentials above the OER onset and reaches its final maximum fraction faster in Fe-KOH (after the 6th CV cycle), whereas it takes 2 more cycles in Fe-free electrolyte. Once the system has reached a dynamic equilibrium, the change of the Ni^3+^ component fraction with the applied potential is more significant in Fe-KOH, reversibly changing between 0.4 and 1 (0.6 to 1 in Fe-free KOH).

Overall, the more significant changes of the component fractions during potential cycling and the lower change in the oxidation state indicate a facilitated transformation between the component present at low potentials and the highly OER active Ni^3+^ component, resulting in an improved OER performance of Ni-MOF* in Fe-containing KOH.

### OER activity and stability of Ni metal organic compound (Ni-MOC*) electrocatalyst upon Fe uptake

As the MOF crystal framework is irreversibly collapsing during OER while the electronic and local structure of the Ni metal centers transform depending on the applied potential, an amorphous Ni species with outstanding OER activity is formed (Ni-MOF*). Based on those results, a simple method to synthesize this highly OER active component without electrochemical decomposition and restructuring of a pristine metal organic framework was developed to facilitate industrial application.^38^ This scalable synthesis resulted in the evolution of a novel amorphous Ni component referred to as Ni metal organic compound (Ni-MOC*, characterizations in Fig. S12, ESI†).^38^

**Fig. 3:**
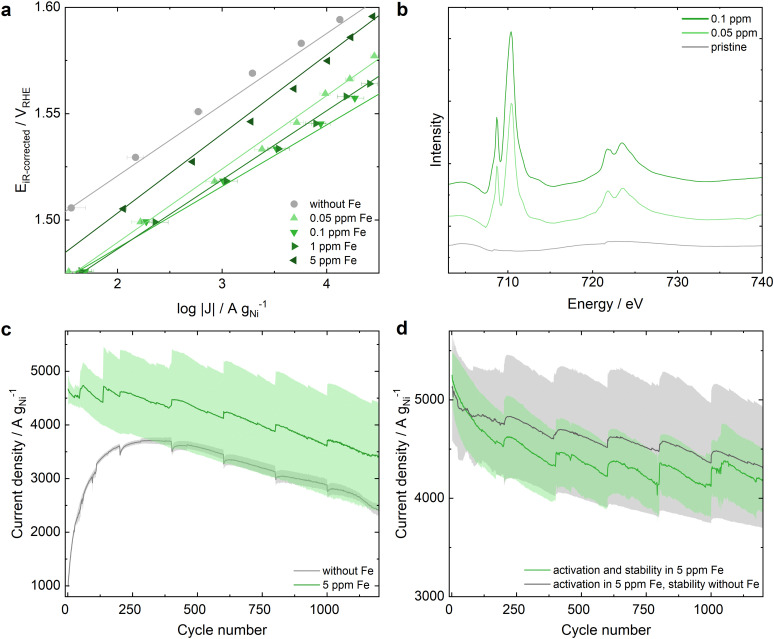
Electrochemical Activity and Stability of Ni-MOC* upon Fe incorporation from Fe-containing 0.1 M KOH electrolyte. The details of the electrochemical measurement protocols are specified in the ESI.† (a) Tafel plots of Ni-MOC* in KOH electrolyte with varying Fe contamination extracted from chronoamperometric step measurements from 1 to 1.7 *V*_RHE_. The error bars present the standard error after averaging 3 measurements. (b) sXAS conducted at the Fe L edge, studying pristine Ni-MOC* and the electrocatalyst after electrochemical testing in 0.05 and 0.1 ppm Fe containing electrolyte. (c) Stability tests of Ni-MOC* in Fe-free and 5 ppm Fe contaminated KOH. (d) Activation of Ni-MOC* in 5 ppm Fe containing KOH, followed by stability testing in Fe containing (green) and Fe-free (grey) KOH.

This material outperforms Ni-MOF-74 – after 25 CVs for equilibration (Fig. S13, ESI†) – in Fe-free electrolyte with a 24% lower Tafel slope and an OER activity of approximately 750 A g_Ni_^−1^ at 1.55 *V*_RHE_. This outstanding activity can be increased further by the incorporation of Fe into the electrolyte resulting in a maximum increase by a factor of 20 with 0.1 ppm Fe (Fig. 3a). Increasing the Fe ratio in the electrolyte above 0.1 ppm decreases the OER activity, similar as observed by Chung *et al.*^29^ with the maximum Fe surface coverage of NiO_*x*_H_*y*_. Additionally, diffusion incorporation of Fe without electrochemical reaction was tested for 5 ppm Fe, resulting in no significant improvement of the OER activity compared to the measurement in clean KOH. Hence, it can be concluded that electrochemical Fe incorporation accelerates a favorable Fe uptake into Ni-MOC* (Fig. S14, ESI†).

Analyzing the trend of the increased OER activity with increasing Fe content in KOH, the pristine Ni-MOC* sample and the samples after OER in 0.05 ppm and 0.1 ppm Fe were studied with sXAS at the Fe L edge. In Fig. 3b the increased Fe L edge with increasing Fe ratio in KOH is detected and corresponds to the reference material Fe_2_O_3_,^36,37^ indicating the presence of Fe^3+^ on the surface of Ni-MOC*. Therefore, the sXAS results of the Fe L edge of Ni-MOF-74 and Ni-MOC* are very similar for the respective Fe ratios in 0.1 M KOH electrolyte.

Focusing on the stability measurement of Ni-MOC*, it outperforms Ni-MOF-74 with an increased activity of factor 3 in Fe-free KOH and can enhance its superior activity even further in Fe-KOH (Fig. 3c). However, the favorable stabilization effect of Ni-MOF-74 upon an initial activation in Fe KOH followed by stability testing in clean KOH is minimal for Ni-MOC* (further protocol variations are depicted in Fig. S15, ESI†). Even with this optimized protocol, there is still a significant decrease in OER activity of more than 10% after 1200 stability test cycles for Ni-MOC*. Nevertheless, the current density at 1.6 *V*_RHE_ of Ni-MOC* at the end of the stability test is still approximately 1800 A g_Ni_^−1^ higher than for Ni-MOF*, emphasizing the superior OER performance of Ni-MOC* compared to Ni-MOF*.

### Transformations of Ni-MOC* upon electrocatalysis in Fe containing electrolyte

**Fig. 4:**
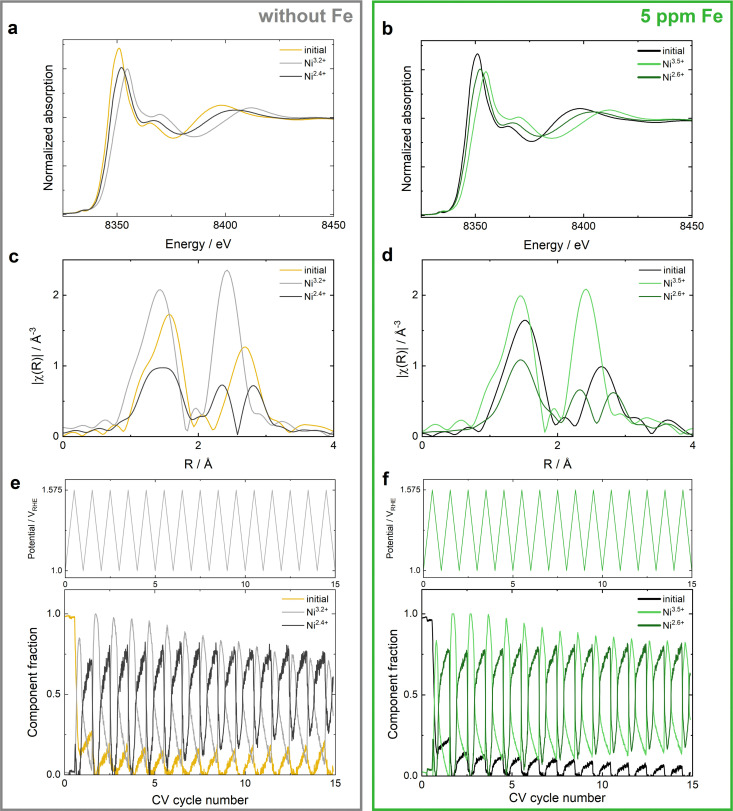
Operando hXAS measurements of Ni K edge of Ni-MOC* during OER in clean (left) and 5 ppm Fe contaminated (right) 0.1 M KOH. (a) and (b) XANES of Ni-MOC* extracted from MCR analysis of 15 CV cycles. (c) and (d) Fourier-transformed k^2^-weighted EXAFS components extracted by the MCR analysis. (e) and (f) Change of the component fractions of the three MCR components during 15 CV cycles.

The local structure and electronic transformations of the Ni center in Ni-MOC* were studied with operando hXAS applying the same electrochemical protocol as described above for Ni-MOF-74. The beam stability and the stability in 0.1 M KOH electrolyte are analyzed with Fig. S16 (ESI†). Fig. 4a–d show the MCR components extracted from the overall operando measurements of Ni-MOC* in Fe-free and Fe containing KOH, the oxidation state determination and the lack of fit are stated in Fig. S17 and S18 (ESI†). A direct comparison of the MCR components of Ni-MOF-74/Ni-MOF* and Ni-MOC* is depicted in Fig. S19 and S20 (ESI†). The monitored Fe induced changes focusing on XANES and EXAFS are comparable to those of Ni-MOF-74 during OER, indicating similar transformations being responsible for the increased OER activity in Fe containing KOH. The incorporation of Fe from the electrolyte results in a less reduced Ni^2.6+^ component at low potentials and a more oxidized Ni^3.5+^ component at high potentials (Fig. 4a and b). The EXAFS (Fig. 4c and d) shows a more pronounced splitting of the first shell of Ni^3.5+^ and a less pronounced one for Ni^2.6+^ in comparison to the MCR components extracted at high and low potentials obtained in Fe-free KOH. The minimized splitting of the first shell of the Ni^2.6+^ component indicates more uniform Ni–O bonds and hence, a less distorted oxygen environment. In contrast to the observations for Ni-MOF-74, the development of the component fractions during CV cycling (Fig. 4e and f) exhibits no significant differences between the operation in Fe-KOH and in Fe-free KOH. However, the changes of the Ni^3.5+^ and Ni^2.6+^ components with applied potential in both electrolytes are more significant for Ni-MOC* than for the MCR components of Ni-MOF-74, indicating facilitated transformations between the two components as a favorable aspect for the higher OER activity of Ni-MOC*. In addition, the average bulk Ni oxidation state of 3.5 in Ni-MOC* at high potentials indicates the stabilization of Ni^4+^ species.^39^ Overall, the Fe uptake results in higher oxidations states of both MCR components and a facilitated transformation between those, thereby boosting the OER activity.

### Application of Ni-MOC* in AEM-WE operation with Fe contaminations

**Fig. 5:**
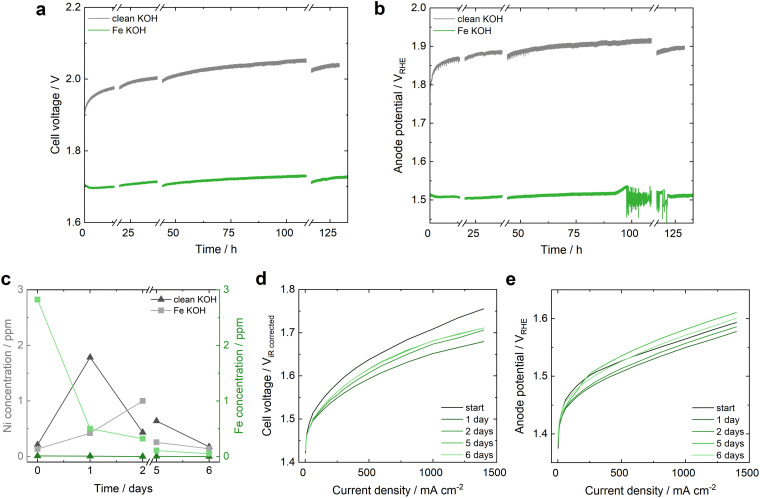
Performance of Ni-MOC* anode in AEM-WE in Fe-free (grey) and 3 ppm Fe contaminated (green) 1 M KOH. (a) and (b) Overall cell voltage and anode potential recorded during constant current operation at 500 mA cm^−2^. For polarization curve measurements, the operation had to be stopped, indicated by the breaks in both graphs. The increase of the anode potential at 94 h and the following fluctuations until 121 h are related to a drying out of the central reversible hydrogen reference electrode compartment that was stabilized by KOH hydration after hour 121. (c) Ni and Fe concentration in the KOH electrolyte monitored by ICP-OES. (d) and (e) Polarization curve measurements of the *iR*-corrected overall cell voltage and the anode potential plotted as the average of the up- and downwards scans.

The Fe incorporation into Ni-MOC* was investigated in a single-cell anion exchange membrane water electrolyzer operated at 0.5 A cm^−2^ and 60 °C in 3 ppm Fe containing 1 M KOH. SEM images of the electrode before and after AEM-WE operation are depicted in Fig. S21 (ESI†). The setup was previously reported by Leuaa *et al.*^40^ and is described in detail in the ESI.† Initially, the electrolyte was contaminated with 3 ppm of Fe and was then recirculated throughout the measurement without additional Fe spiking. The cell voltage and anode potential obtained during more than 130 h of testing in Fig. 5a and b can be compared to the operation with a pure Ni felt anode in Fig. S22 (ESI†). Implementing a Ni-MOC*/Ni felt anode, a stable overall performance was achieved for more than 130 h with a remarkable improvement of 200 mV in overall cell voltage and 300 to 400 mV in anode potential compared to the operation in Fe-free 1 M KOH (Fig. 5a and b). The Fe contamination of the 1 M Fe-free KOH was determined by ICP-OES to be max. 0.02 ppm. While the initial activation in clean KOH results in a worse performance, an initial improvement is observed in Fe containing KOH. This performance increase within the first days correlates to the decrease of the Fe ratio in the electrolyte detected by inductively coupled plasma optical emission spectrometry (ICP-OES) after the first day of operation (Fig. 5c), indicating Fe uptake of the Ni-MOC* catalyst. The anode performance is very stable at a remarkable anode potential of 1.51 *V*_RHE_ at start and end of operation, even though the Fe uptake probability from the electrolyte is minimized in the last days, as the Fe ratio within the KOH decreases to approximately 0.05 ppm at day 6. Additionally, the Ni dissolution detected is smaller during the tests in Fe contaminated KOH than in Fe-free KOH, showing a possible stabilization of the Ni electrode in Fe containing KOH. The polarization curves of the overall cell voltage and the anode potential, reported as the average of the up- and downwards current density scan (Fig. 5d and e), present an initial activation of the overall cell voltage, related to the cathode performance increase on day 1 (Fig. S23, ESI†) and a very stable performance of the anode with *circa* ±16 mV at 1.4 A cm^−2^ within 6 days (±50 mV in Fe-free KOH, Fig. S24, ESI†).

## Conclusions

Herein, the activity increase of Ni-MOF-74 and an amorphous Ni metal organic compound upon Fe incorporation from the electrolyte is investigated by unveiling the electronic and structural transformations of both electrocatalysts during OER in Fe containing KOH electrolyte. XRD analysis revealed an amorphization of Ni-MOF-74 and time resolved operando hXAS measurements show that in the presence of Fe in the electrolyte, both catalysts exhibit higher oxidation states of the Ni metal centers at low applied potentials. Additionally, a higher Ni oxidation state during OER was determined for Ni-MOC*. Based on the information provided by the operando hXAS results for Ni-MOF-74 and Ni-MOC* in the absence/presence of Fe traces in the electrolyte, we conclude that the outstanding activity in Fe-KOH is enabled by the stabilization of the more oxidized, highly OER active Ni centers upon Fe uptake and a facilitated transformation of those Ni centers upon changes of the applied potential. These results showcase the importance of high Ni oxidation states and amorphous, convertible components for increased OER activities that can be achieved by Fe incorporation. Complemented by the promising stability during continuous 130 h AEM-WE operation at 0.5 A cm^−2^, this work bridges fundamental operando studies with industrial application, offering an impressive perspective for the utilization of natural Fe contaminations in AEM and alkaline water electrolyzers to optimize the performance of Ni-based OER electrocatalysts.

## Author contributions

J. L. conducted the experimental work, the data analysis and wrote the paper. T. R. synthesized the studied catalysts. A. H. C. assisted in operando hXAS measurements, data analysis and results discussion. C. B. and T. H. assisted in sXAS measurements, data analysis and results discussion. F. L. B., M. R. K. and C. C. assisted in AEM-WE single cell test measurements, data analysis and results discussion. E. M., J. G. and M. W. performed RDE and ICP measurements of both catalysts. E. F., provided project ideas and contributed by project supervision, results discussion and financial contribution. M. R. and T. J. S. contributed to project supervision and results discussion. All authors contributed to manuscript revision.

## Data availability

Data for this article, including all raw data for the depicted figures are available at Materials Cloud Archive at https://doi.org/10.24435/materialscloud:q2-rd.

## Conflicts of interest

There are no conflicts to declare.

## Supplementary Material

EY-003-D4EY00250D-s001
