# Supplementary material for: The role of Fe incorporation into Ni-MOF-74 derived oxygen evolution electrocatalysts for anion exchange membrane water electrolysis
Source: EES Catal. 2025 Feb 4;3(3):505–14. doi: 10.1039/d4ey00250d (PMC11791620; doi:10.1039/d4ey00250d)
Supplement: EY-003-D4EY00250D-s001 [file EY-003-D4EY00250D-s001.pdf]

Supplementary Information (SI) for EES Catalysis.
This journal is © The Royal Society of Chemistry 2025

Supplementary information

The role of Fe incorporation into Ni-MOF-74 derived oxygen evolution electrocatalysts for anion exchange membrane water electrolysis

Julia Linke[a], Thomas Rohrbach[a], Adam Hugh Clark[b], Camelia Borca[b], Thomas Huthwelker[b], Fabian Luca Buchauer[c], Mikkel Rykær Kraglund[c], Christodoulos Chatzichristodoulou[c], Eibhlin Meade[a], Julie Guehl[a], Mateusz Wojtas[a], Marco Ranocchiari[a], Thomas Justus Schmidt[a,d], Emiliana Fabbri*[a]

[a] *PSI Center for Energy and Environmental Science, 5232 Villigen PSI, Switzerland.*
[b] *PSI Center for Photon Science, 5232 Villigen PSI, Switzerland.*
[c] *Department of Energy Conversion and Storage, Technical University of Denmark, Kongens Lyngby, Denmark.*
[d] *Institute of Molecular Physical Science, ETH Zürich, 8093 Zürich, Switzerland*
* Corresponding author

Experimental Section

Materials

0.1 M KOH and 1 M KOH electrolytes were prepared using ultrapure Milli-Q water (18.2 MΩcm) and 99.99% pure KOH pellets (Sigma Aldrich; certificate of analysis states: no Fe impurity, but 70.8 ppm of other trace metals (Ba, Ca, Cs, Li, Rb) and 225.1 ppm Na impurity) and are referred to as "Fe-free" and "clean" KOH. $Fe(NO_3)_2 \cdot 9\ H_2O$ for Fe contamination of the KOH was bought from Sigma Aldrich. Dimethylformamide (DMF) from Fisher Scientific (HPLC grade, 99.5% purity), *tert*-butyl methyl ether (TBME) from Honeywell (99.5% purity) and ethanol from VWR chemicals were used as solvents for the catalyst syntheses. 2,5-Dihydroxyterephtalic acid from Fluorochem (98% purity) was recrystallized from ethanol:water 1:1. NiO nanopowder (18 nm size, cubic) was bought from Nanografi, $Ni(NO_3)_2 \cdot 6\ H_2O$ purchased from Sigma Aldrich and $Ni(acac)_2$ from Acros Organics / Thermo Scientific. All chemicals were used without further purification.

Methods

Synthesis of Ni-MOF-74. Ni-MOF-74 was produced applying the slightly modified reported microwave reactor MOF-74 synthesis method[1, 2]. 3002 mg of 2,5-Dihydroxyterephtalic acid was added to a solution of dimethylformamide (DMF), ethanol and water (1:1:1) to form a stock solution (molarity 0.101 mol l^{-1}). 30 mL of solvent solution (DMF/ethanol/water = 1:1:1) and 30 mL of stock solution were added with 2731 mg of $Ni(NO_3)_2 \cdot 6\ H_2O$ to a 100 mL EasyPrep vessel. To obtain a brown suspension, the mixture was stirred in a microwave reactor at 130 °C for 1.5 h. The resulting powder was filtered using a membrane filter with 30 mL of each of the following solvents: DMF, hot water, ethanol and *tert*-butyl methyl ether (TBME). A yield of approx. 1.55 g of yellow powder was gained after the Ni-MOF-74 powder was dried overnight at 60 °C in a vacuum oven. Additionally, the powder was heat-treated for 16 h at 150 °C to ensure complete evaporation of the solvents used during synthesis. Afterwards, successful synthesis was confirmed by powder X-ray diffraction (PXRD), attenuated total reflection infrared spectroscopy (ATR-IR), Brunauer-Emmett-Teller (BET) surface area analysis and transmission electron microscopy (TEM). Inductively coupled plasma - optical emission spectrometry (ICP-OES) was used to confirm the Ni metal content of 35 wt.-% in the synthesized MOF-74 powder.

Synthesis of Ni-MOC*. A green solution was obtained after dissolving 6.03 g of $Ni(acac)_2$ (23.47 mmol) in 600 mL ethanol at 50 °C. Afterwards, 150 mL ethanol containing 1500 mg of 2,5-dihydroxyterephtalic acid (7.57 mmol) was added and the mixture was stirred at 50 °C for 60 min, producing a light green suspension. Membrane filtration and washing with 150 mL of ethanol at 50 °C and 400 mL of ethanol at room temperature was done to extract the yellowy green powder. It was dried in a vacuum oven at 60 °C overnight resulting in 2.65 g of brown powder. The successful synthesis was confirmed by PXRD, ATR-IR, BET surface area analysis and TEM. ICP-OES was used to confirm the Ni metal content of 35 wt.-% in the synthesized powder.

Electrochemical measurements. An RDE setup with a modulated speed rotator (Pine Research Instrumentation) set to 900 rpm and a multichannel potentiostat (VMP-300, BioLogic Science Instruments) was used to study the electrochemical activity and stability of the as-synthesized catalysts in a 3-electrode cell setup. 0.1 M KOH saturated with synthetic air (5.6 purity, PanGas AG Switzerland) was used as electrolyte and as the filling of the Hg/HgO reference electrode (RE61AP, ALS). This RE was calibrated against a polycrystalline Pt disc inserted in H_2-saturated electrolyte, resulting in all RDE measurement results being reported vs. RHE. In addition, the results were normalized by the catalyst mass defined by the electrocatalyst ink that is dropcasted as 2.5 µL on a polished glassy carbon disc (SIGRADUR®, HTW GmbH) to achieve a thin-film RDE environment[3, 4]. The ink is produced as a solution of 7 mL Milli-Q water and 28 mL isopropanol (99.9%, Sigma Aldrich) with 70 µL Na^+-exchanged Nafion (5 wt.-%, Sigma Aldrich) containing 5 mg of catalyst. The Ni-MOF-74 ink shaken properly instead of sonicated to ensure that the pristine MOF-74 structure is tested[5, 6], whereas the Ni-MOC* ink was sonicated for 1 min directly before dropcasting. The counter electrode is a flame-annealed gold mesh. For assessing the OER activity and stability, two different protocols were applied. The **OER activity protocol** started with equilibrating the catalyst's

surfaces during 25 CVs between 1.0 and 1.7 V_{RHE} applying a scan rate of 10 mV s^{-1}. Afterwards, 15 chronoamperometric (CA) steps of 60 s from 1.2 V_{RHE} to 1.7 V_{RHE} were conducted. Potentiostatic electrochemical impedance spectroscopy (PEIS) was conducted before the CVs and after the CA steps to extract the ohmic drop for iR correction. The **OER stability measurement protocol** included PEIS measurements at 1.2 V_{RHE} before and after the following protocol: 3 CVs from 1 V_{RHE} to 1.6 V_{RHE} (scan rate of 50 mV s^{-1}) followed by 200 CA steps of 1 V_{RHE} and 1.6 V_{RHE}, respectively (10 s per potential step). This procedure was repeated 6 times, adding up to 1200 CA steps at 1 V_{RHE} and 1.6 V_{RHE}. The result of the stability test is reported as the mass-normalized, averaged steady-state current obtained at 1.6 V_{RHE} plotted against the CA cycle number. All RDE measurement results are iR-corrected and the displayed error bars for all electrochemical results represent the standard deviation of three measurements.

Material characterizations. Cu Kα radiation was applied for PXRD measurements using a Bruker D8 Advance diffractometer equipped with a Lynxeye XE detector in Bragg-Brentano geometry. The data was recorded at a step size of 0.02° from 2Θ = 4° to 2Θ = 40°. Before conducting the nitrogen adsorption experiments for surface characterization (Micromeritics 3Δ Flex), the catalyst was activated at 250 °C for 16 h under vacuum using a Micromeritics VacPrep 061 sample degas system. The gravimetric surface area was calculated with the Brunauer-Emmett-Teller (BET) method. Infrared spectroscopy experiments were conducted with a Bruker Vertex70 spectrometer in the attenuated total reflection mode with a Deuterated alanine doped Tri-Glycine Sulphate ATR detector by accumulating 32 scans at a spectral resolution of 4 cm^{-1}. For the acquisition of the TEM images, a cold field emission gun (FEG) at 200 kV was used as the electron beam source (JEM-ARM200F NEOARM, JEOL, Japan). A catalyst with 5 mg catalyst in 3 mL milli-Q water was prepared and shaken for 1 min (sonicated for 1 min when preparing Ni-MOC*). Then, 5 µL of the ink was dropcasted onto a carbon-sputtered TEM grid (continuous ultrathin carbon film coated lacey carbon supported copper grid (LC400-CU-CC-25), Sigma-Aldrich, Germany). Afterwards, an additional carbon layer was sputtered on top of the dried catalyst layer on the TEM grid before starting the imaging. ICP-OES was performed using the axial viewing mode of Agilent 5110 ICP-OES. 932 µL of each KOH sample was diluted with 4.027 mL of 5 wt.-% HNO_3 and 40.8 µL of 65 wt.-% HNO_3 to result in a 5 mL ICP sample. 5 mg of each powder sample was digested with the following steps, while heating and vortex mixing the solution in between each of the steps: 0.1 mL of 96 % H_2SO_4, 0.1 mL of 65 % HNO_3, 0.1 mL of 30 % HCl, 0.1 mL of milli-Q water. The resulting solution was filled up to a total volume of 5 mL using 0.2 M HNO_3. Scanning electron microscopy (SEM) was carried out using a Zeiss Merlin SEM.

Synchrotron XAS and XRD data collection and analysis. The XRD measurements were conducted in transmission mode at the Materials Science beamline of the Swiss Light Source (SLS; PSI Switzerland). For the detector position calibration, the refinement of the Au diffraction pattern of an Au-sputtered polyetherimide electrode positioned in the flow cell setup was used. The 25 keV beam was 0.49211 Å calibrated with NIST SRM 660a LaB6 standard and had a diameter of 150 µm (adjusted by use of a pinhole) with 400 mA current. The patterns were collected with a Pilatus 6M area detector. For the operando X-ray absorption spectroscopy (XAS) measurements at the SuperXAS beamline of the SLS at PSI, the catalysts were positioned in a homemade flow cell[7] with a 3-electrode setup. Thereby, the catalyst samples were sprayed as an ink of Milli-Q water and Na^+-exchanged Nafion onto gold-sputtered carbon-coated Kapton foil to act as the working electrode. A black pearl carbon (2000 carbon black from Cabot Corporation) sprayed gold-sputtered carbon-coated Kapton foil counter electrode and a commercial Ag/AgCl RE (3 M NaCl filled, Harvard Apparatus) were used. Before and after each operando measurement, the Ag/AgCl RE was measured versus the Hg/HgO RE described above for conversion into RHE scale. Using a syringe pump with a rate of 0.4 mL min^{-1} the 0.1 M KOH electrolyte was pulled through the flow cell. The electrochemical protocol was applied with a Biologic SP300 potentiostat and tailored to decrease bubble formation and accumulation in order to reduce measurement inaccuracies[8]. The photon beam from the 2.9 T superbend magnet of the SLS was collimated with a Si-coated mirror at 2.9 mrad and monochromatized with a liquid nitrogen cooled channel-cut Si(111) crystal that oscillated at a frequency of 1 Hz[9]. The beam was focused to a spot size of 0.2 mm x 1 mm with a Rh-coated toroidal mirror. The flowcell was positioned between the first and second ionization chamber and the Ni reference foil between the second and

third ionization chamber. All operando measurements were conducted in quick fluorescence mode with a PIPS detector[10], while the ex situ Ni reference pellet measurements were conducted in transmission mode. Calibration, normalization and averaging were conducted with the ProQEXAFS software[11] and the lack of fit for MCR component analysis is listed in Figs. S8, S15. The ex situ soft-XAS (sXAS) measurements were conducted at the low-energy branch line of the Phoenix beamline at SLS. The low-energy branch of the beamline (sourced by an elliptical undulator) utilizes the optics and the planar grating monochromator of the X-treme beamline[12]. The incoming flux of light is derived from the total electron yield (TEY) signal of an Au grid before the sample chamber, in which the samples are studied in a vacuum of 10^{-6} mbar. The detection depth of approximately 10 nm results in a surface-sensitive measurement. All studied samples were deposited as an ink (see RDE ink above) dropcasted on a glassy carbon disk. The disk was attached to the sample holder with conductive carbon tape. For all samples, the same amount of material was tested, and the samples were measured under equal conditions (electrical connection for TEY measurement, range of amplifier, etc.). After performing I0 correction, the signal strength obtained for the Fe L edge is a measure of the amount of Fe in the beam, especially as the shape of the signal is the same for all tested samples, indicating the presence of similar Fe species. The recorded absolute intensity of the Fe edge is significantly different among the tested samples, indicating a clear trend of higher Fe contents in both catalysts after electrochemical testing in KOH with higher Fe contamination.

AEM-WE test setup and procedure. The zero-gap flow cell setup described previously by Leuaa et al.[13] was applied for AEM-WE single-cell testing. To be used as the anode, the Ni-MOC* catalyst was sprayed on 2Ni06-0.20 Ni felts from NV Bekaert SA. The cathode consisted of a commercial Pt/C cathode (0.4 mg_{Pt} cm^{-2}, Johnson Matthey). All electrodes were cut to a diameter of 36 mm resulting in an active electrode area of 10 cm^2. The cell was operated at 0.5 A cm^{-2} and 60 °C (heating applied in the cell housing behind the flow fields) with a Sustainion X37-50RT anion exchange membrane. The membrane was activated according to the procedure stated by Dioxide Materials: Activation in Fe-free 1 M KOH for 8 h, exchange of the KOH solution and further soaking in Fe-free 1 M KOH for more than 24 h. Afterwards the membrane was stored in Fe-free 1 M KOH for up to 8 weeks until it was used for testing. The 1 M KOH electrolyte for the Fe-containing AEM-WE tests was contaminated with 3 ppm of Fe before starting the test. The KOH was recirculated and consumed water was added to the KOH solution automatically throughout the test duration while no further addition of Fe took place. Initially, the cells were conditioned at 0.1 A cm^{-2} for 60 min after increasing the current density stepwise in the following order (each step a max. of 2 min): 10 mA cm^{-2}, 50 mA cm^{-2}, 100 mA cm^{-2}. As a reference test for comparison of the Fe KOH tests, the pure Ni felt without any Ni-MOC* catalyst deposited on it was applied as an anode in a cell with the same batch of commercial Pt/C cathode and of Sustainion X37-50RT membrane, respectively (Figs. S21). Gear pumps were used to pump the electrolyte at a constant flow rate of 25 mL min^{-1} through the anode and cathode compartments. The electrolysis cell employed contains a RE setup discussed in detail as the inactive wire RE setup of Bohn et al.[14]: an additional RHE reference electrode is positioned directly at the membrane in addition to the cathodic and anodic REs in the respective electrolyte supply channels. All reference electrodes in the setup were reversible hydrogen electrodes (RHE, Mini-HydroFlex, Gaskatel), except for a Hg/HgO reference electrode (RE-61AP, ALS Co., Ltd) in the anodic electrolyte channel position. The potentials measured vs. this Hg/HgO RE were converted into RHE scale by the anode potential difference between RHE and Hg/HgO at open circuit voltage. A Biologic SP300 potentiostat was utilized for potentiostatic impedance spectroscopy measurements at 1.2 V for iR-correction. The Fe and Ni content of the KOH electrolyte were monitored by sampling 1 mL of the electrolyte solution at the anodic side after the first 5 min (initial) and at the morning of day 1, 2, 5 and 6. 932 µL of the KOH sample were added to 4.027 mL of 5 wt.-% HNO_3 and 40.8 µL of 65 wt.-% HNO_3 to test the resulting solution using the Agilent Technologies ICP-OES instrument.

Figures

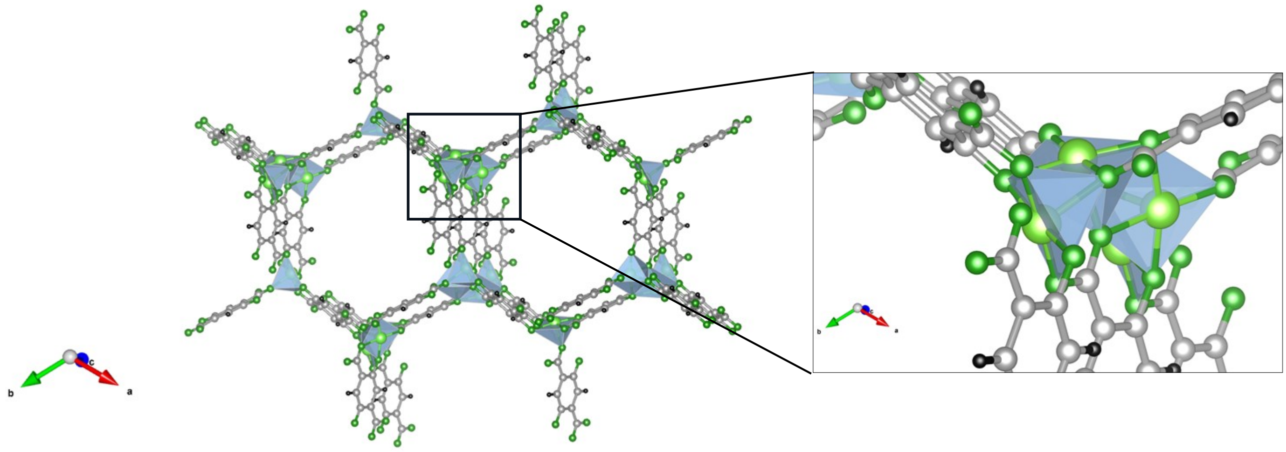

Fig. S1 Crystal structure of Ni-MOF-74 with a zoom into the local structure of the Ni centres in Ni-MOF-74. The Ni atoms are shown in light green, oxygen atoms in dark green, hydrogen atoms in black and carbon atoms in grey.

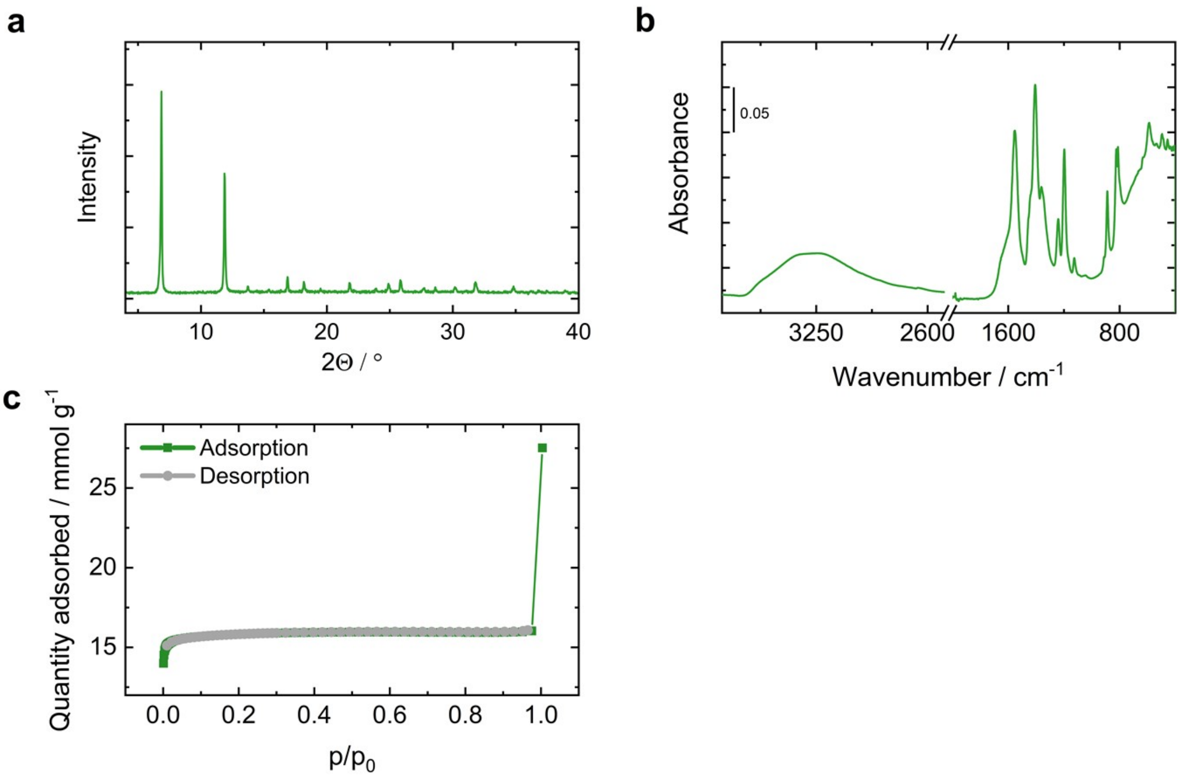

Fig. S2 Characterization of the pristine Ni-MOF-74 powder after successful synthesis. (a) PXRD of the obtained powder after the final heat treatment step after successful synthesis. (b) ATR-IR of the successfully synthesized material. (c) BET surface area analysis of Ni-MOF-74.

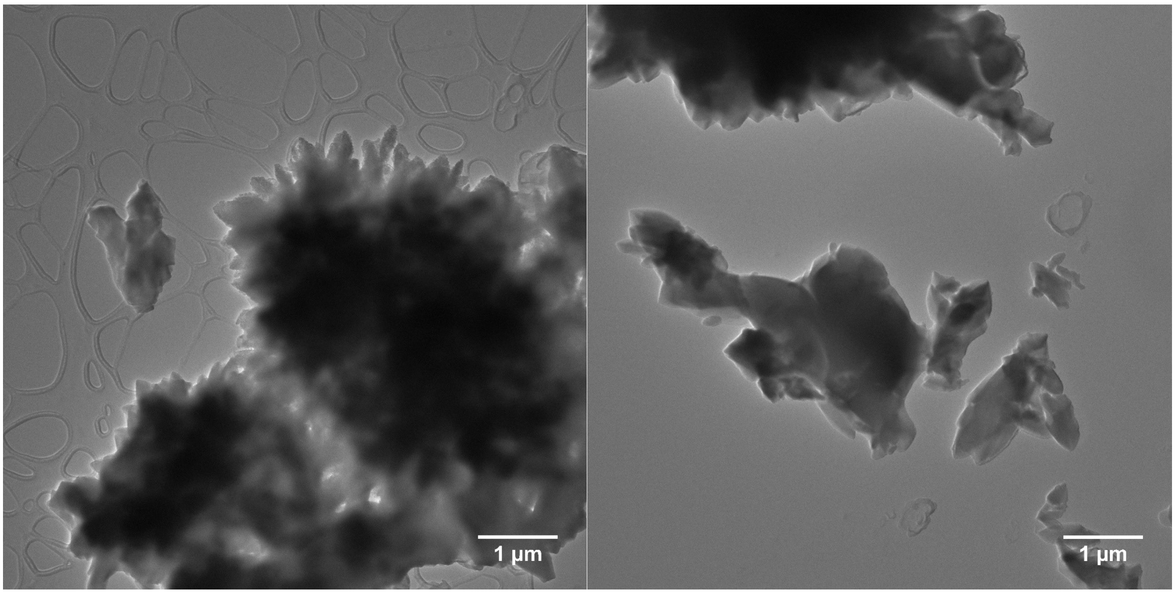

Fig. S3 Transmission electron microscopy images of Ni-MOF-74.

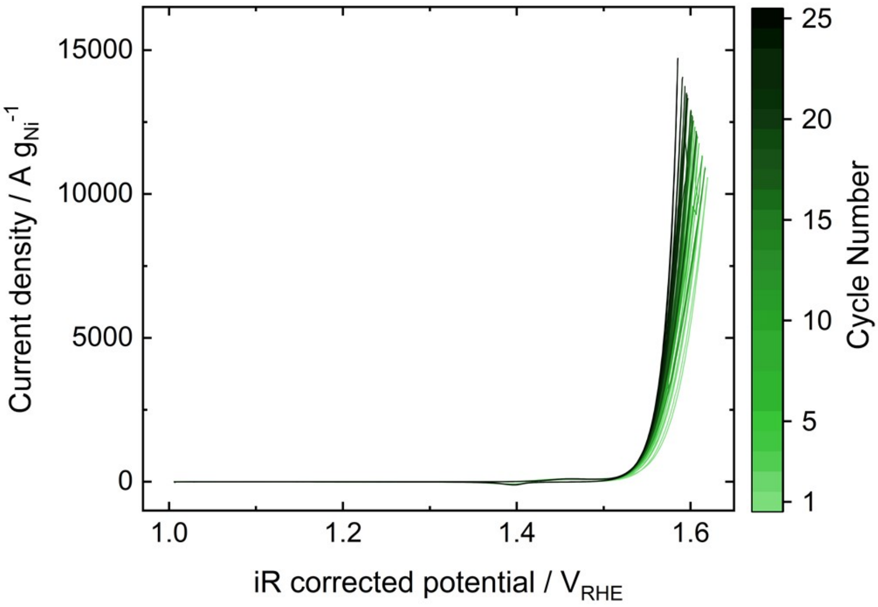

Fig. S4 25 CVs recorded in 5 ppm Fe contaminated 0.1 M KOH from 1 to 1.7 V_{RHE} with a scan rate of 10 mV s^{-1} to equilibrate the OER performance of Ni-MOF-74 before the chronoamperometric step measurements for the Tafel plot extraction.

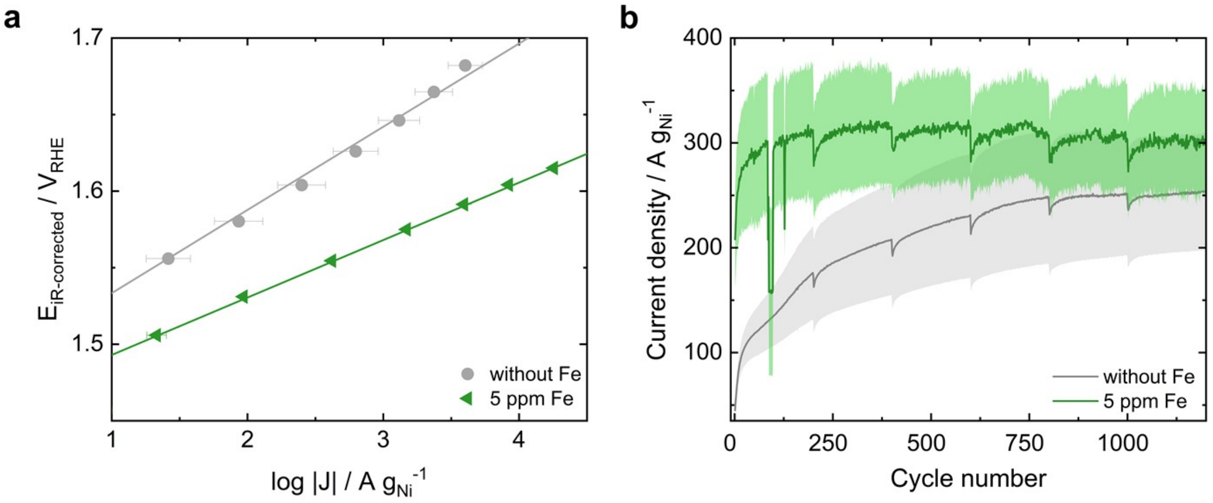

Fig. S5 The activity and stability of NiO in clean and 5 ppm Fe contaminated 0.1 M KOH. (a) Tafel plot depicting the OER activity of NiO: The highest activity of 330 A g_{Ni}^{-1} at 1.55 V_{RHE} with a Tafel slope of 38 mV dec^{-1} is reached in Fe-containing KOH electrolyte. (b) Stability of NiO in clean 0.1 M KOH and in 5 ppm Fe containing 0.1 M KOH.

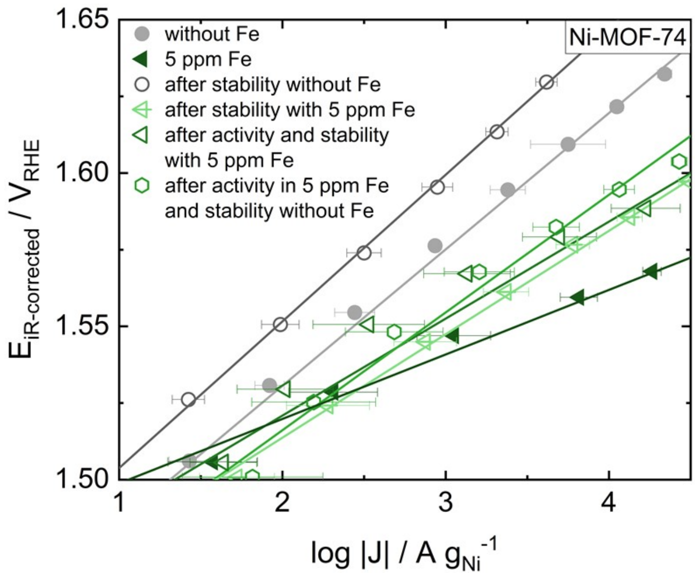

Fig. S6 Activity of Ni-MOF-74 after variations of stability and activity measurements with and without Fe contamination in the KOH electrolyte. The filled grey circle and dark green triangle are the activities obtained during activity testing, all other measurements are variations of activity and stability test protocols as specified in the legend. The highest activity and lowest Tafel slope were achieved after stability testing in 5 ppm Fe contaminated KOH without initial activation, resulting in an activity of approximately 2700 A g_{Ni}^{-1} at 1.55 V_{RHE} and a Tafel slope of 21 mV dec^{-1}.

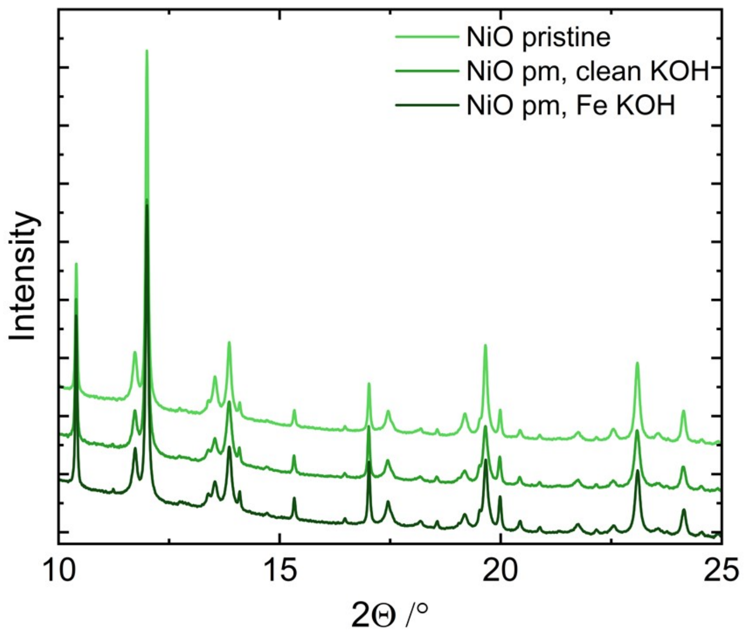

Fig. S7 XRD measurements of NiO before and after electrochemical testing in clean and 5 ppm Fe containing 0.1 M KOH. No changes in the crystal structure of NiO (and the peaks of the Au support used for electrode preparation) were detected.

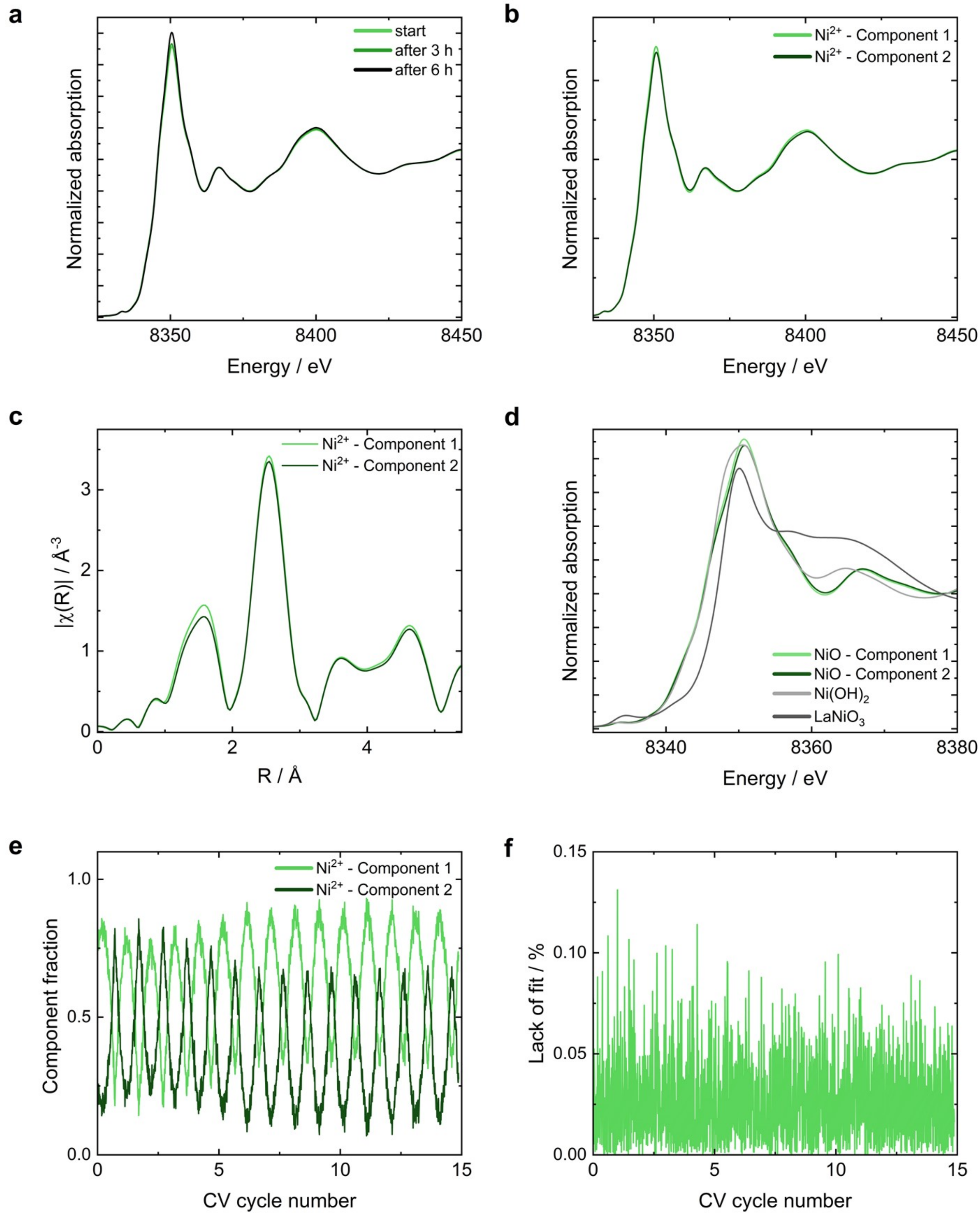

Fig. S8 hXAS measurements of NiO. (a) Electrolyte stability of NiO tested by soaking the electrode in 0.1 M KOH. (b) XANES of the two detected MCR components. (c) EXAFS of both MCR components of NiO. (d) Oxidation state determination of both MCR components: The same Ni oxidation state of 2+ is extracted. (e) Changes of the component fractions of both MCR components during 15 CV cycles. (f) Lack of fit (LOF) of the MCR analysis of NiO.

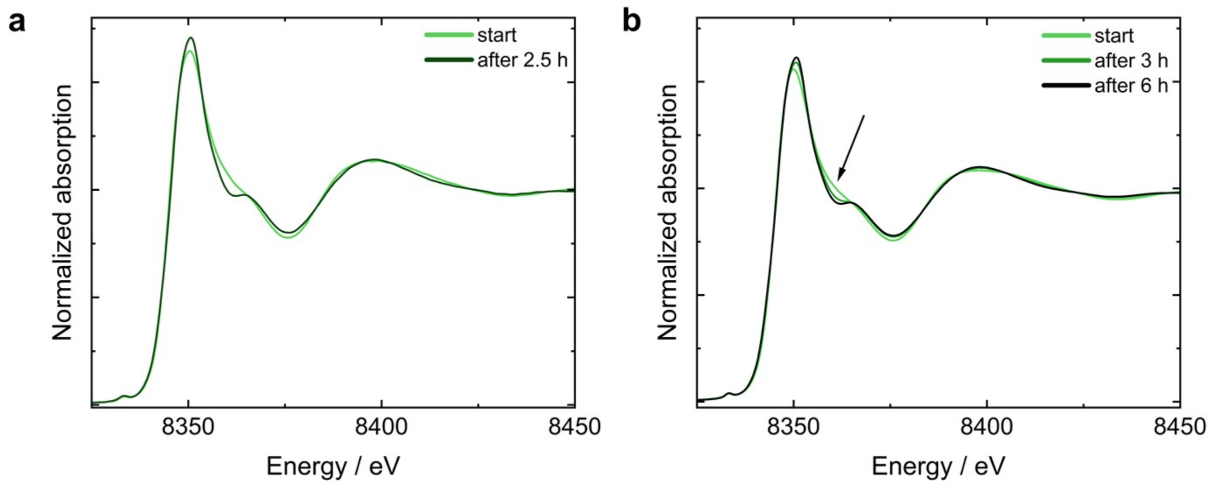

Fig. S9 Analysis of the beam stability (a) and electrolyte stability (b) of Ni-MOF-74 in Fe-free KOH. (a) The beam stability was tested by continuous XAS measurements conducted in the flow cell with 0.1 M KOH present and without electrochemical testing of Ni-MOF-74. (b) The electrolyte stability was tested by soaking Ni-MOF-74 in 0.1 M KOH without applied beam nor electrochemical testing. The comparison of (a) and (b) clarifies that the changes monitored in (a) result from the contact of Ni-MOF-74 with 0.1 M KOH electrolyte and hence, there are no significant beam-induced changes.

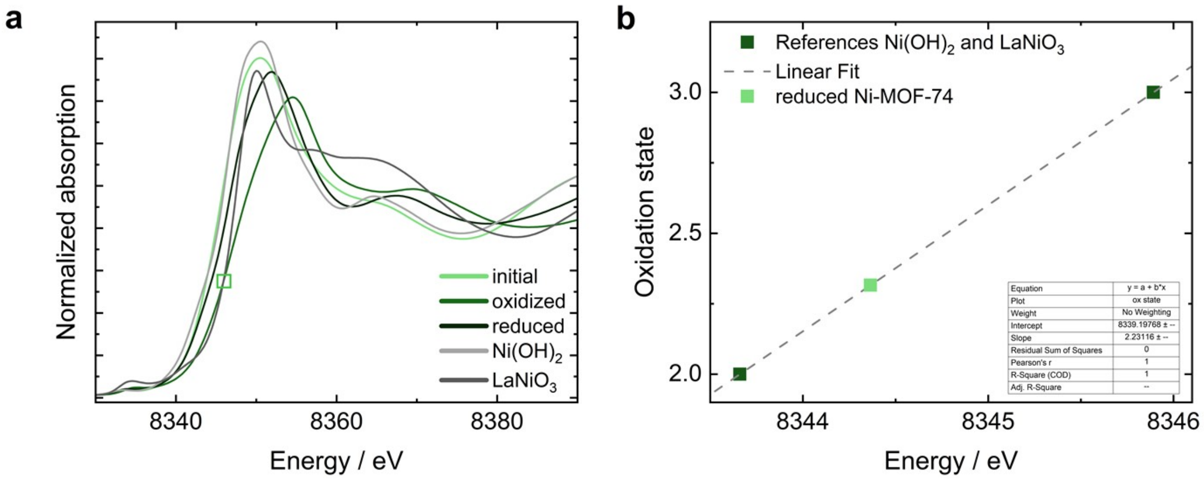

Fig. S10 Oxidation state determination of Ni-MOF-74 based on XANES comparison with reference materials.

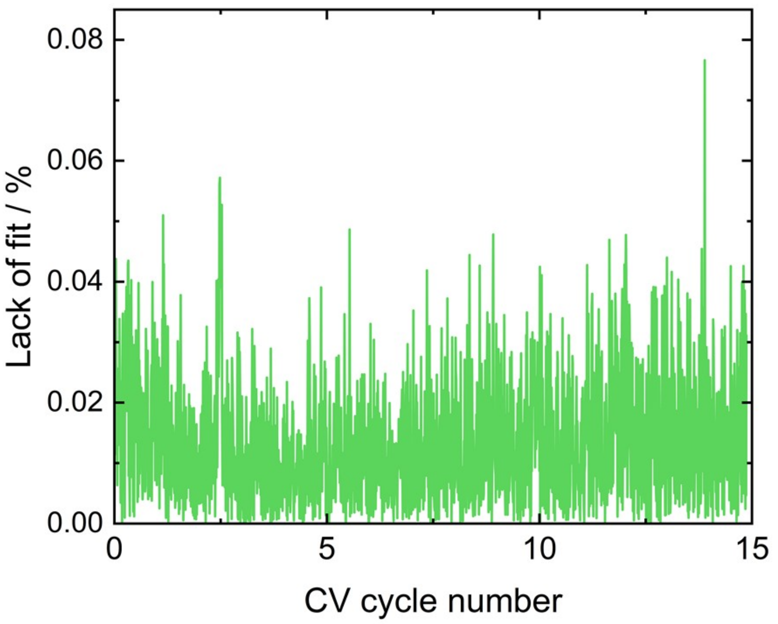

Fig. S11 Lack of fit (LOF) of MCR analysis of Ni-MOF-74.

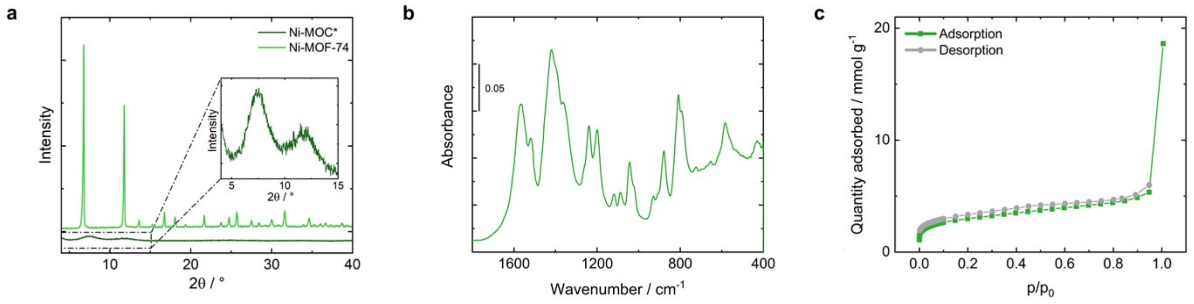

Fig. S12 Characterization of the pristine Ni-MOC* powder after the successful synthesis: (a) PXRD of pristine Ni-MOC* compared to Ni-MOF-74, (b) ATR-IR of the successfully synthesized material, (c) BET surface area analysis of Ni-MOC*.

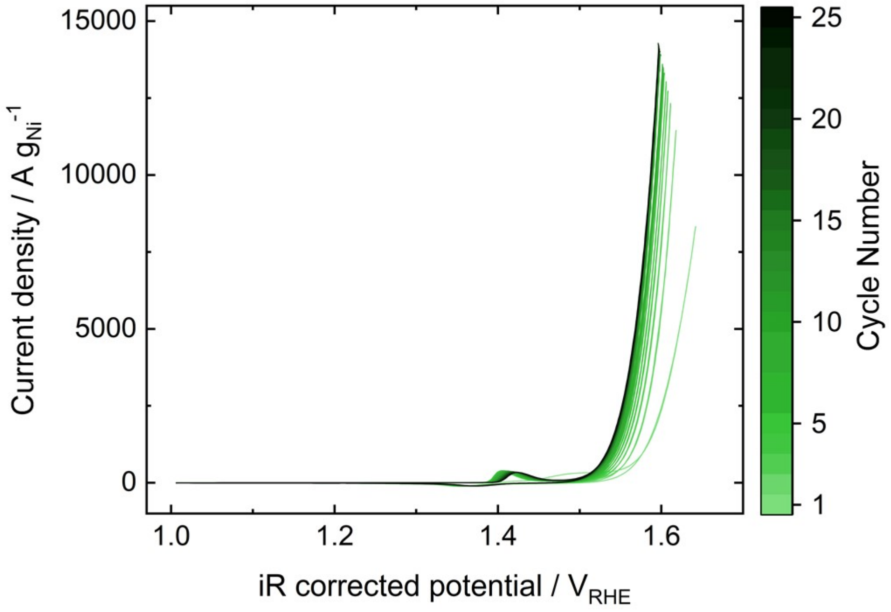

Fig. S13 25 CVs recorded in 5 ppm Fe contaminated 0.1 M KOH from 1 to 1.7 V_{RHE} with a scan rate of 10 mV s^{-1} to equilibrate the OER performance of Ni-MOC* before the chronoamperometric step measurements for the Tafel plot extraction.

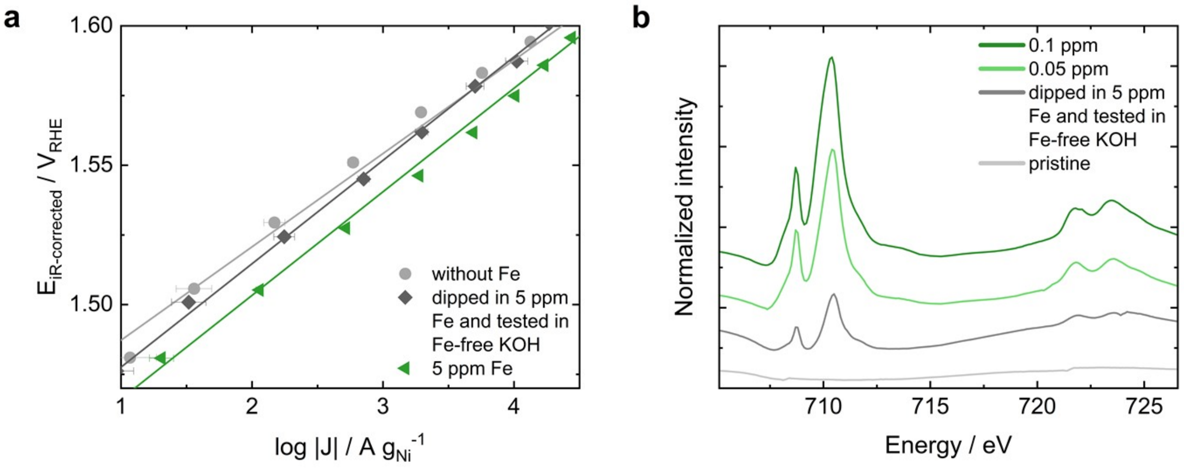

Fig. S14 Analysis of the OER activity after diffusion incorporation of Fe into Ni-MOC*. (a) The activity was tested in Fe-free KOH after dipping the electrode for 1 min in 5 ppm Fe containing 0.1 M KOH electrolyte. The comparison to the Fe-free electrochemical activity and the activity in 5 ppm Fe contaminated KOH shows no significant improvement of the OER activity upon diffusion-incorporation of Fe. (b) sXAS of the Fe L edge depicting the Fe uptake upon diffusion incorporation vs. electrochemical incorporation after OER. A significant Fe diffusion incorporation is shown; however, the Fe uptake is less effective then upon electrochemical reaction in Fe-containing KOH. Hence, electrochemical Fe incorporation results in a higher Fe uptake and an increased OER activity.

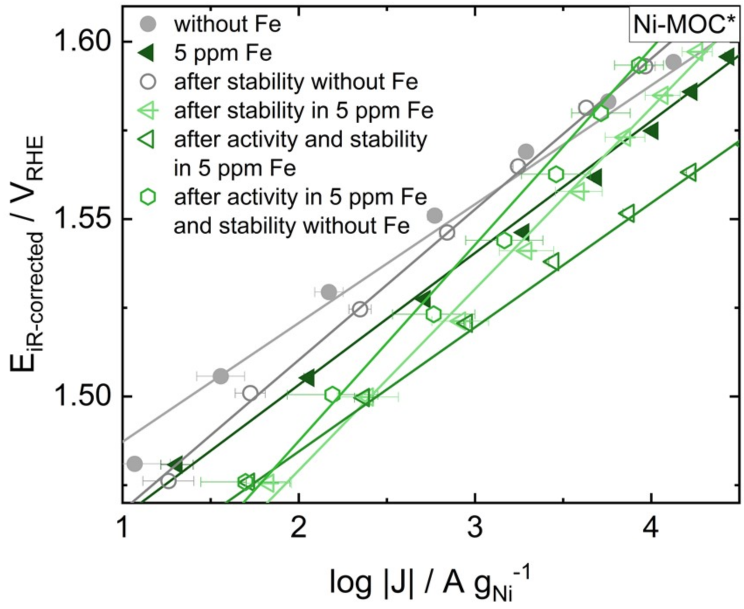

Fig. S15 OER activity of Ni-MOC* after variations of stability and activity measurements with and without KOH. The filled grey circle and dark green triangle are the activities obtained during activity testing, all other measurements are variations of activity and stability test protocols as specified in the legend. Comparing the stated measurement protocol variations, the highest activity is obtained after conducting activation and stability measurement in 5 ppm Fe containing 0.1 M KOH electrolyte, resulting in approximately 1800 A g_{Ni}^{-1} at 1.55 V_{RHE} and a Tafel slope of 37 mV dec^{-1}. This activity is

significantly lower than during activation testing in 0.1 ppm Fe containing electrolyte, indicating the importance of an optimized Fe uptake into Ni-MOC* for maximal OER activity.

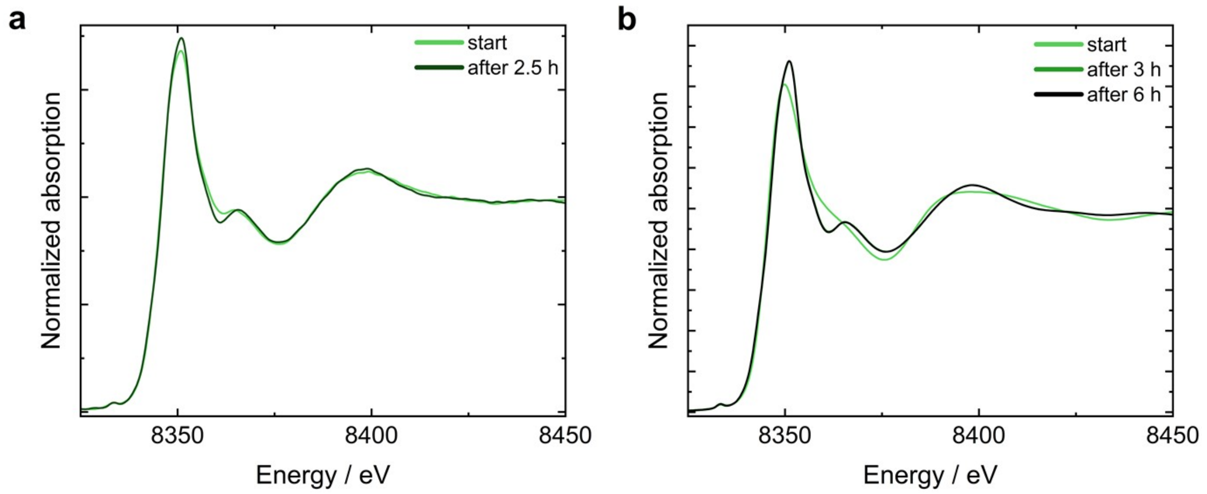

Fig. S16 Analysis of the beam stability (a) and electrolyte stability (b) of Ni-MOC* in Fe-free KOH. (a) The beam stability was tested by continuous XAS measurements conducted in the flow cell with 0.1 M KOH present and without electrochemical testing of Ni-MOC*. (b) The electrolyte stability was tested by soaking Ni-MOC* in 0.1 M KOH without applied beam nor electrochemical testing; the curves for 3 h and 6 h are identical, indicating that most of the electrolyte-induced changes occur within the first 3 h. The comparison of (a) and (b) clarifies that the changes monitored in (a) result from the contact of Ni-MOC* with 0.1 M KOH electrolyte and hence, there no significant beam-induced changes were recorded.

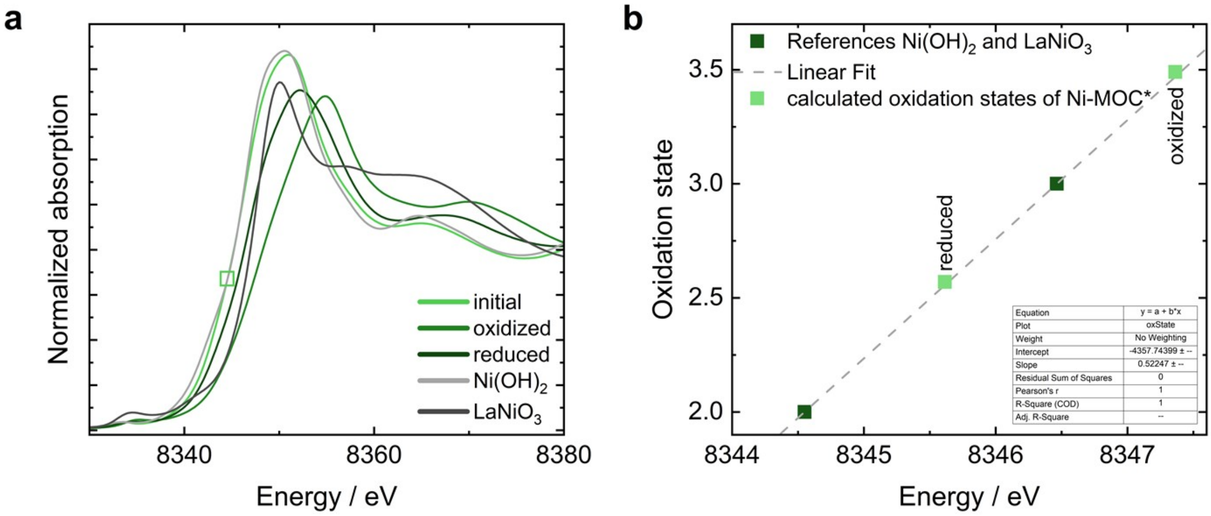

Fig. S17 Oxidation state determination of Ni-MOC* based on the comparison with reference materials.

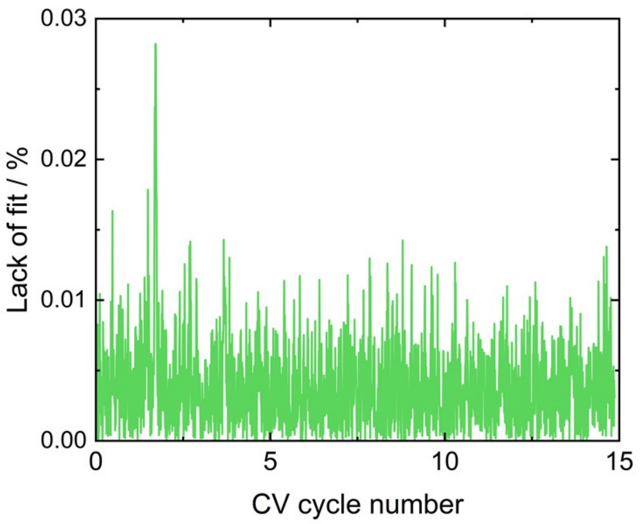

Fig. S18 Lack of fit (LOF) of the MCR analysis of Ni-MOC*.

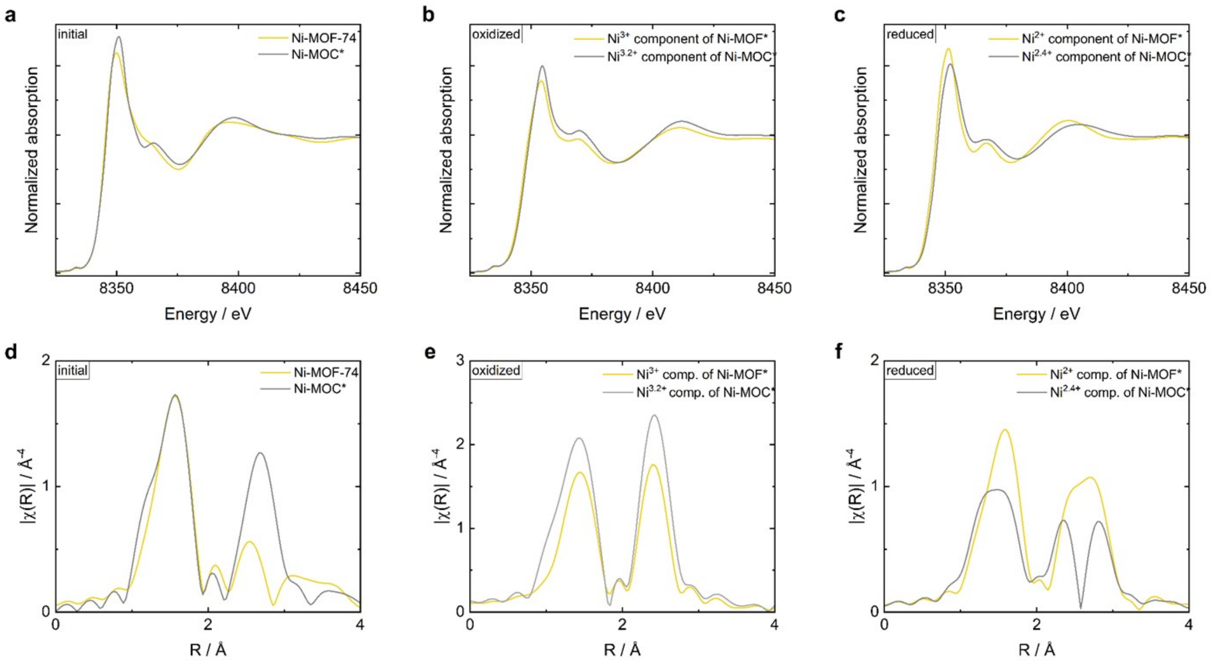

Fig. S19 Direct comparison of the operando XAS results of Ni-MOF-74/Ni-MOF* and Ni-MOC* obtained during testing in Fe-free 0.1 M KOH.

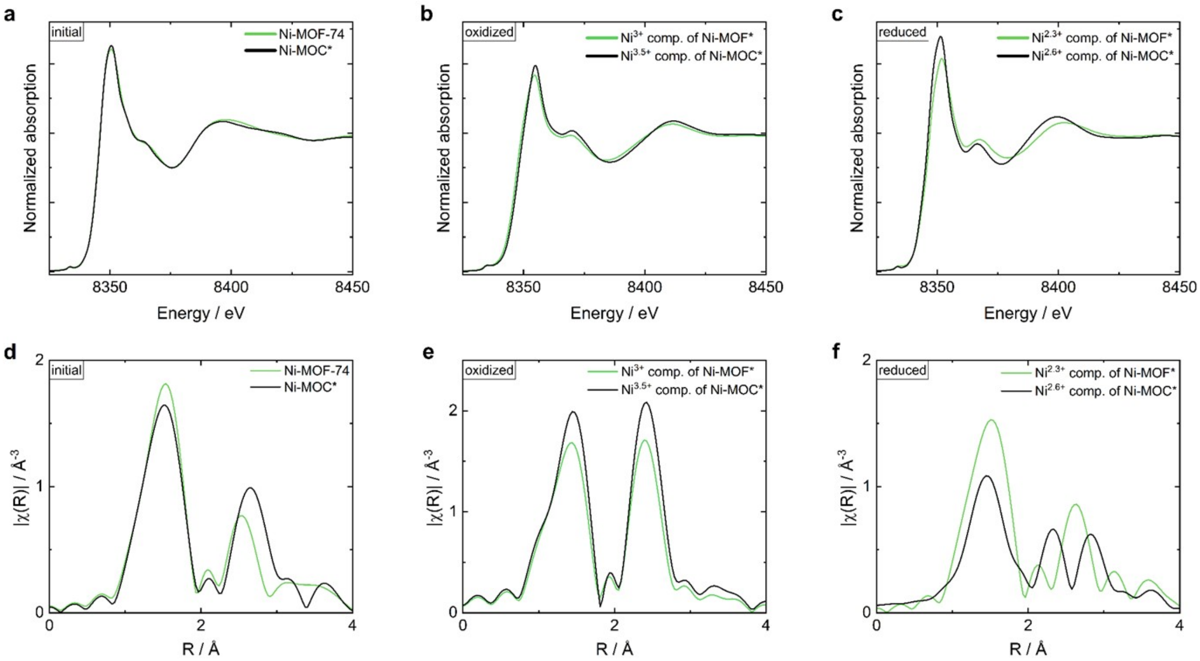

Fig. S20 Direct comparison of the operando XAS results of Ni-MOF-74/Ni-MOF* and Ni-MOC* obtained during testing in 5 ppm Fe containing 0.1 M KOH.

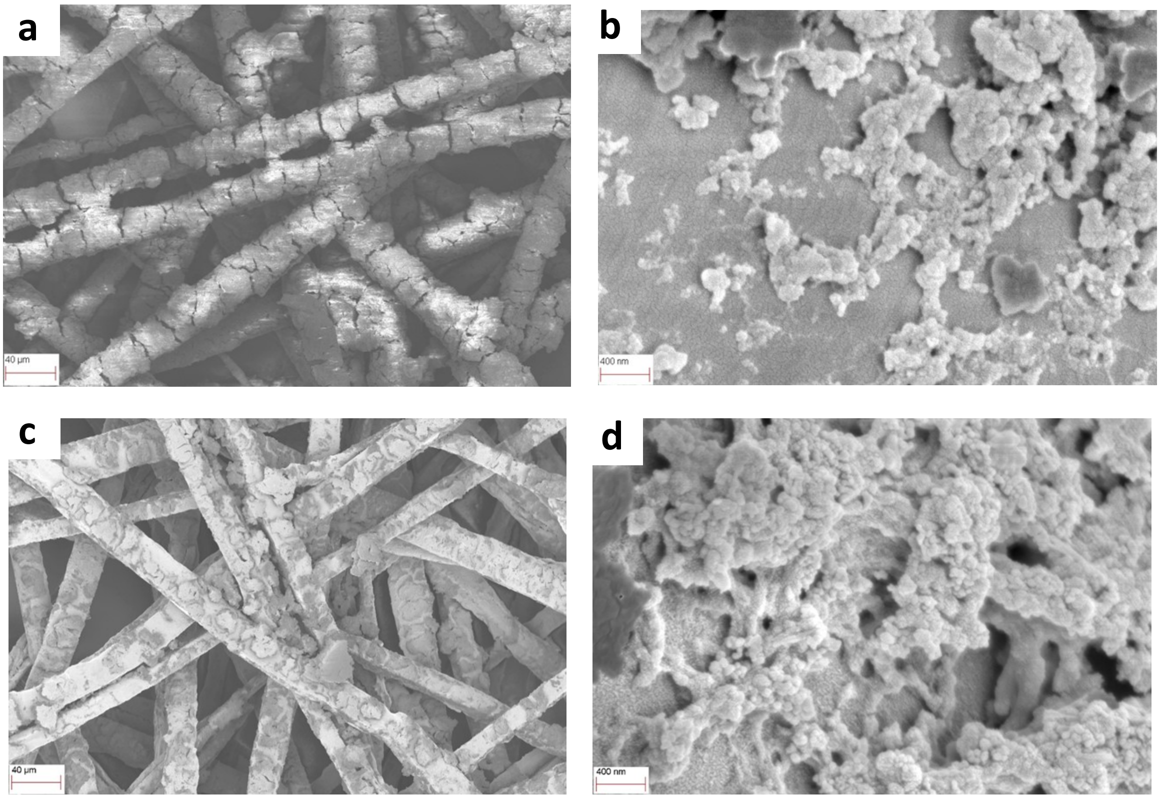

Fig. S21 SEM images of Ni-MOC*/Ni felt anode before (a-b) and after (c-d) testing in AEM-WE with 3 ppm Fe containing 1 M electrolyte.

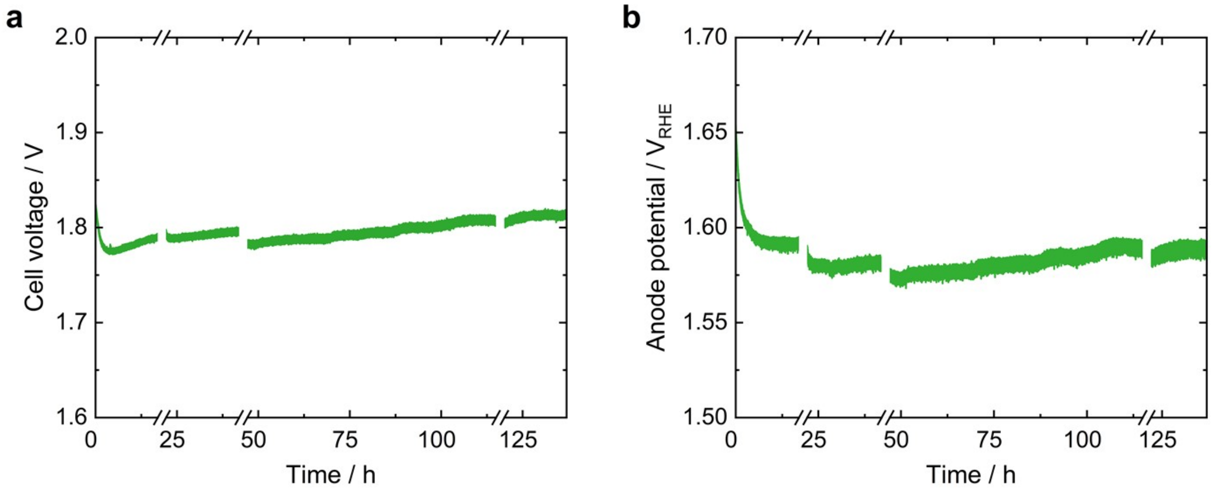

Fig. S22 Cell voltage and anode potential monitored during testing of a pure Ni felt anode with a commercial Pt/C cathode and a Sustainion X37-50RT membrane in 3 ppm Fe contaminated vs. in clean 1 M KOH operated at 500 mA cm^{-2} and 60 °C. For polarization curve measurements, the operation had to be stopped, indicated by the breaks in both graphs.

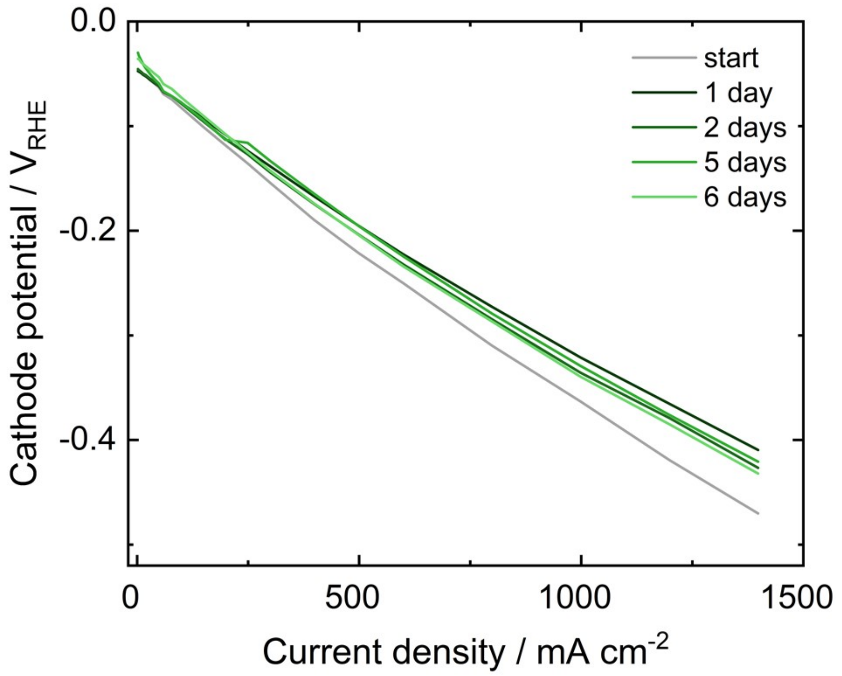

Fig. S23 Cathode potential polarization curves obtained during AEM-WE with a Ni-MOC*/Ni felt anode, a commercial Pt/C cathode and a Sustainion X37-50RT membrane in Fe-contaminated 1 M KOH.

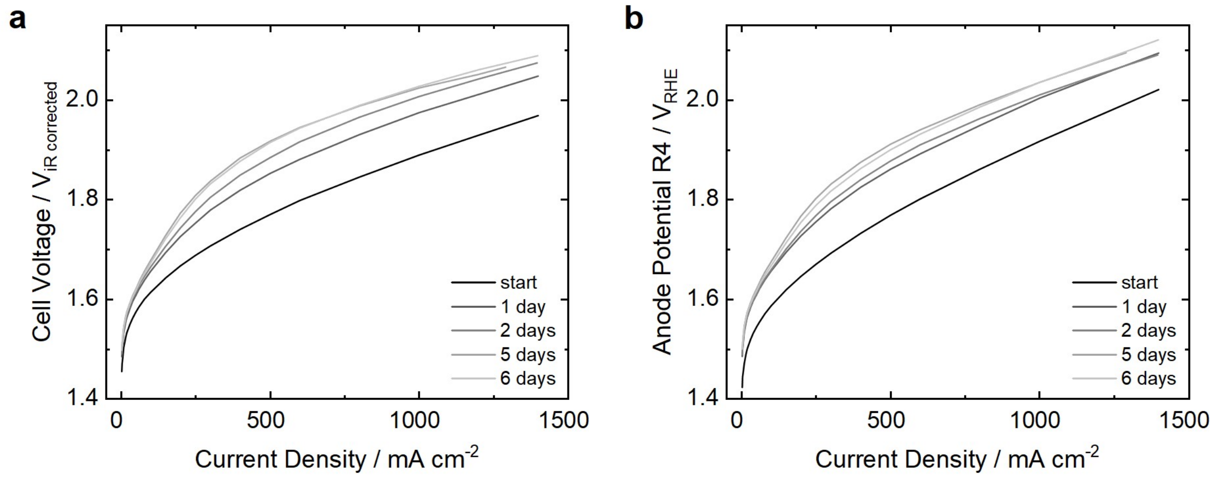

Fig. S24 Polarization curves obtained during AEM-WE test of Ni-MOC*/Ni felt anode and commercial Pt/C cathode with a Sustainion X37-50RT membrane in clean 1 M KOH.

References

1. P. Gaumann, D. Ferri, D. Sheptyakov, J. A. van Bokhoven, P. Rzepka and M. Ranocchiari, *J Phys Chem C Nanomater Interfaces*, 2023, **127**, 16636-16644.
2. P. Gaumann, T. Rohrbach, L. Artiglia, D. Ongari, B. Smit, J. A. van Bokhoven and M. Ranocchiari, *Chemistry*, 2023, **29**, e202300939.
3. T. J. Schmidt, H. A. Gasteiger, G. D. Stab, P. M. Urban, D. M. Kolb and R. J. Behm, *Journal of The Electrochemical Society*, 1998, 2354.
4. T. J. Schmidt and H. A. Gasteiger, in *Handbook of Fuel Cells*, 2010, DOI: 10.1002/9780470974001.f203024.
5. Q. Jiang, C. Zhou, H. Meng, Y. Han, X. Shi, C. Zhan and R. Zhang, *Journal of Materials Chemistry A*, 2020, **8**, 15271-15301.
6. U. Khan, A. Nairan, J. Gao and Q. Zhang, *Small Structures*, 2022, **4**, 2200109.
7. T. Binninger, E. Fabbri, A. Patru, M. Garganourakis, J. Han, D. F. Abbott, O. Sereda, R. Kötz, A. Menzel, M. Nachtegaal and T. J. Schmidt, *Journal of The Electrochemical Society*, 2016, **163**, H906-H912.
8. N. Diklić, A. H. Clark, J. Herranz, J. S. Diercks, D. Aegerter, M. Nachtegaal, A. Beard and T. J. Schmidt, *ACS Energy Letters*, 2022, **7**, 1735-1740.
9. O. Muller, M. Nachtegaal, J. Just, D. Lutzenkirchen-Hecht and R. Frahm, *J Synchrotron Radiat*, 2016, **23**, 260-266.
10. A. H. Clark, P. Steiger, B. Bornmann, S. Hitz, R. Frahm, D. Ferri and M. Nachtegaal, *J Synchrotron Radiat*, 2020, **27**, 681-688.
11. A. H. Clark, J. Imbao, R. Frahm and M. Nachtegaal, *J Synchrotron Radiat*, 2020, **27**, 551-557.
12. C. Piamonteze, U. Flechsig, S. Rusponi, J. Dreiser, J. Heidler, M. Schmidt, R. Wetter, M. Calvi, T. Schmidt, H. Pruchova, J. Krempasky, C. Quitmann, H. Brune and F. Nolting, *J Synchrotron Radiat*, 2012, **19**, 661-674.
13. P. Leuaa, M. R. Kraglund and C. Chatzichristodoulou, *Electrochimica Acta*, 2023, **470**, 143306.
14. L. Bohn, J. Kieninger, S. J. Rupitsch, C. Klose, S. Vierrath and J. Disch, *Adv Sci (Weinh)*, 2024, DOI: 10.1002/advs.202402095, e2402095.
